# An iterative approach to statistical optimization of exopolysaccharide produced by fermentation of *Aureobasidium pullulans*

**DOI:** 10.1016/j.btre.2025.e00914

**Published:** 2025-08-15

**Authors:** Venessa Dsouza, Goldee Thoidingjam, Abdelrahman Saleh, Michael Zavrel

**Affiliations:** a Technical University of Munich, Germany, TUM Campus Straubing for Biotechnology and Sustainability, Bioprocess Engineering, Uferstraße 53, D-94315 Straubing, Germany; b Munich Institute of Integrated Materials, Energy and Process Engineering, Technical University of Munich, Garching, Germany; c Catalytic Research Center, Technical University of Munich, Garching, Germany

**Keywords:** Exopolysaccharide, Statistical optimization, *Aureobasidium pullulans*, Pullulan

## Abstract

Exopolysaccharides are biopolymers with wide-ranging industrial applications. To substitute fossil-based by bio-based, biodegradable polymers, exopolysaccharide production needs to become much more efficient. Pullulan, produced by *Aureobasidium pullulans*, is popular for its unique properties like film-formation, adhesiveness, biodegradability, etc. This study aims at demonstrating the optimization strategy on pullulan production using statistical optimization iteratively. The process followed a systematic approach including One Factor at A Time (OFAT) and Design Of Experiments (DOE). OFAT confirmed sucrose as carbon source, pH 6 and 4 days of incubation were optimum. Iterative Plackett-Burman Design (PBD) and Response Surface Methodology (RSM) further demonstrated interactions between significant media components and incubation conditions. The final pullulan titer was increased 6.34-fold to 113.5 ± 3.5 g/L of EPS, the highest titer achieved for this strain in shake flasks. Future investigations into fed-batch strategies and scale-up studies can be explored to further improve titers and reduce overall production costs.

## Highlights

• Innovative approach combining rational process development with iterative statistical optimization of medium components and process conditions.
• 6.34 fold increase of pullulan production by fermentation.
• Highest titer of 113.5 g/L for pullulan production from *Aureobasidium pulllulans* WT strain.

## 1 Introduction

Exopolysaccharides (EPS) are extracellular polysaccharides, usually excreted into the culture medium by bacteria and fungi under certain conditions [1]. They are biopolymers with high molecular weights, and their chemical structures constitute mainly of sugars, proteins, humic substances, lipids, or extracellular DNA [2]. EPS cultures may become very viscous, with some varieties also solidifying into gels. EPS can be part of the cellular capsule or also be observed as loose slime secreted externally. The polymeric nature of these polysaccharides makes them an attractive alternative to synthetic plastics and petroleum-based rheology modifiers [3].

However, production of EPS depends on the strain used, composition of the media, physiological conditions, and the substrate adopted with the operating conditions [4]. Yang et al. [5], confirmed that the best carbon source for EPS production is specific to the strain. Media composition and external conditions are the main factors affecting the production of EPS [6]. This suggests that optimization of media is also strain specific and cannot be extrapolated directly from literature.

*Aureobasidium pullulans* (*A. pullulans*) is a fungus with a yeast-like appearance. It was named “black yeast” because it produces a black melanin pigment [7]. It is widely known for its production of the EPS pullulan. Pullulan has a chemical structure consisting of α−1,6-maltotriose units linked together by α−1,6 glycosidic bonds, each maltotriose unit has three glucose units linked by α−1,4 glycosidic bonds [8].

These systemic alpha linkages provide pullulan its unique elasticity and water solubility [9]. Some of the unique physical properties of pullulan include its adhesive ability, fiber-forming capability, and its ability to form transparent thin films and biodegradable substances [10]. Pullulan is used as a food additive, adhesive, and packaging material, and is also an ingredient in the cosmetic industry [[11], [12], [13]]. Recent applications include drug delivery, low-viscosity filler, and scaffolds [14]. Gaikwad et al. [15], discussed a novel application of pullulan for corrosion mitigation.

According to Zhang et al. [16], the production of metabolites like pullulan and the cell division of *Aureobasidium* spp. require an extensive amount of energy. However, there are a few issues with its fermentative production, including (i) the melanin pigment production; (ii) inhibitory effects coming from high substrate concentrations; and (iii) high broth viscosity and associated costs [17]. The applications were restricted, because wild-type strains of *A. pullulans* overcoming these issues were not readily available. To utilize its versatile industrial applications, an economic and optimized media formulation for pullulan production is required.

Various organisms and different strain variants exhibit selective nutrient requirements affecting their growth and production of products. Therefore, screening of factors that are significant to the responses is important to further optimize the effect of the factors [18]. PBD is a two-level design, particularly helpful in finding the main effects while assuming other interactions to be insignificant. When comparing significant major effects, i.e., in the absence of interactions, the observed effect of one factor may be overestimated or underestimated by other factors [19]. PBD can be used as a starting point for DOE, and it should be used to determine the list of experiments to be conducted. PBD belongs to “screening designs” as it helps to screen out the non-significant factors from all considered factors [20].

An additional design is necessary, because PBD only considers the main effects and ignores other interactions between the factors. Box & Wilson [21], provided the first description of Central Composite Design (CCD) or RSM. Several operational factors and their combinative effects affect the responses of a process. To analyze these effects and their interactions, statistical optimization strategies applying RSM within the DOE, is a sophisticated tool for data interpretation [22]. According to Gupte & Kulkarni [23], RSM uses multiple optimization phases and can be completed in three simple steps: first, experiments are designed to screen for factors, then the path of most inclined rise or drop is found, and last, a quadratic regression model is fitted and optimized using the canonical regression analysis method. Representing the titer as a surface plot is one of the key RSM inputs [24].

**Fig. 1:**
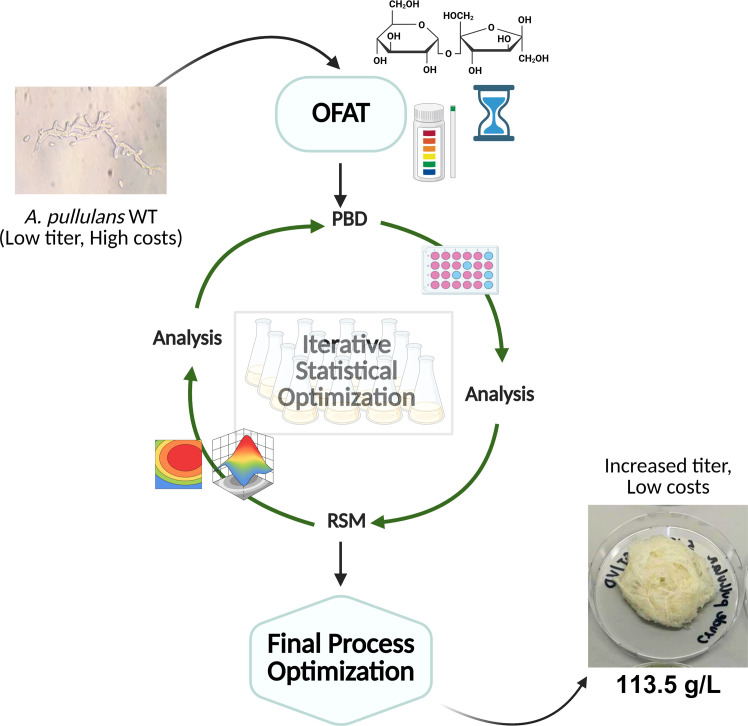
The iterative optimization process applied in this study. Created in BioRender. Dsouza, V. (2025) https://BioRender.com/zlyauvn.

The current investigation aimed at optimizing the media and incubation conditions for pullulan production using OFAT analysis followed by an iterative statistical optimization using *A. pullulans PpKM-3* (DSM 3042) in shake flasks. The last trial involved the optimization of all factors (media components and conditions) at once, testing an integrative design. The study followed the Design-Build-Test-Learn (DBTL) cycle, an iteratively used loop that helps achieve a design satisfying the desired outcome, in this study, a high titer [25]. Fig. 1 illustrates this iterative optimization process.

## 2 Materials and methods

### 2.1 Materials

#### 2.1.1 Strain

The strain *Aureobasidium pullulans* (DSM 3042) was purchased from the Leibniz Institute DSMZ-German Collection of Microorganisms and Cell Cultures GmbH.

#### 2.1.2 Chemicals

Glucose, Sucrose, Fructose, Xylose, Yeast Extract (YE), Ammonium Sulphate ((NH_4_)_2_SO_4_), di-Potassium Hydrogen Phosphate (K_2_HPO_4_), Magnesium Sulphate (MgSO_4_), Sodium Chloride (NaCl), Ethanol, Acetone, Glycerol, Sulphuric Acid (H_2_SO_4_) and Sodium Hydroxide (NaOH)were purchased from Carl Roth GmbH. Pullulan standard was purchased from Fisher Scientific. Cane Molasses was purchased from Nortem Chem S.A., through Amazon.de.

#### 2.1.3 Software

The DOE add-in from OriginLab was used to design the runs and analyze data for all experiments. BioRender was used to create process related figures. OPUS 8.8.4 software was used to analyze the FTIR spectrum.

### 2.2 Methodology

#### 2.2.1 Production and extraction of EPS

**Fig. 2:**
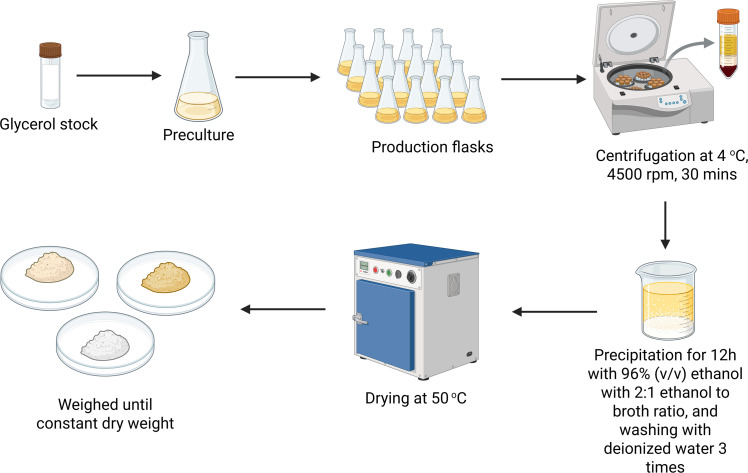
Downstream process for extraction of pullulan. Created in BioRender. Dsouza, V. (2025) https://BioRender.com/7l0v09f.

The strain received from DSMZ was revived according to the instructions provided by DSMZ and inoculated in 100 mL of Media 90 [26], incubated at 28 °C, 140 rpm for 7 days in an Eppendorf Innova S44i incubator with a shaking diameter of 2.5 cm and stored in 2 mL cryovials containing 500 µL of culture and 500 µL of glycerol solution (50 % v/v) at −80 °C for further use. The preculture was prepared in a 500 mL Erlenmeyer flasks containing 100 mL Media 90, adjusted to pH 5.6 using 37 % H_2_SO_4_ and 1 M NaOH, inoculated with the preserved glycerol seed stock and incubated at 28 °C, 140 rpm for 7 days. One seed stock was always used for inoculation of 100 mL Media 90 as a standard for preculture preparation, and a final cell density of 8.5 g/L was achieved in the preculture after 7 days. The production of EPS was carried out in 100 mL flasks containing 20 mL production medium, where the concentrations of components, the pH and the inoculum volume was varied throughout the experiments of the current study (Appendix A). The EPS was harvested and purified following the method of Lee et al. [27], with slight modification. The process for extraction is as depicted in Fig. 2. Further purification was done by washing with 5 mL acetone, followed by 10 mL distilled water to remove other small proteins and extracellular molecules.

#### 2.2.2 OFAT

One Factor at A Time analysis was conducted to test the influence of different carbon sources, pH and incubation period for production of pullulan. The media components kept constant throughout OFAT were: 0.2 % (w/v) YE, 0.02 % (w/v) MgSO_4_, 0.5 % (w/v) K_2_HPO_4_, 0.06 % (w/v) (NH_4_)_2_SO_4_ and 0.1 % (w/v) NaCl. The carbon sources tested were glucose 5 % (w/v), sucrose 5 % (w/v), fructose 5 % (w/v), xylose 5 % (w/v), cane molasses 5 % (v/v) and glycerol 5 % (v/v). After selecting the carbon source, the pH experiment testing a range of pH (2–10) was conducted to select the optimum pH. The incubation period experiment involved harvesting the product each day for 8 days. For OFAT analysis, the inoculum volume, incubation temperature and speed were kept constant at 10 %, 28 °C and 140 rpm respectively.

#### 2.2.3 Statistical optimization

Statistical optimization was done as an iterative process in contrast to the usually followed procedure. This involved 3 trials, where different conditions and media components were varied in each trial to identify the global optimum. A standard strategy starting with the PBD and RSM was used to conduct all 3 trials, while changing the ranges of variables in each trial based on the results of the preceding trial. The 1st trial (T1) consisted of PBD and RSM for media components followed by a PBD and RSM for pH and incubation period. The 2nd trial (T2) consisted of a CCD based on the PBD for media from T1 with increased ranges. It also consisted of a PBD and RSM testing increased ranges for incubation parameters from T1. T3 was employed to test an integrated effect of media components and incubation parameters together in one PBD and RSM.

The media consisted of sucrose, YE, MgSO_4_, K_2_HPO_4_, (NH_4_)_2_SO_4_ and NaCl, with concentrations varying in each trial, the ranges of which are mentioned specific to each experiment in Appendix A. The temperature and speed were constant at 28⁰C and 140 rpm. The pH and incubation period were varied between trials (Appendix A).

The PBD was based on first-order polynomial model equation employed for the analysis of the responses where *Y* indicated the titer:

(1) $$Y=\beta_{0}+\beta_{1}A+\beta_{2}B+\beta_{3}C+\beta_{4}D+\beta_{5}E+\beta_{6}F$$

(β_0_ is the intercept, β_1,2,3,4,5,6_ represent the predictor's coefficients and *A, B, C, D, E, F* represent the independent factors).

The CCD was fitted into a polynomial model described as follows:

(2) $$Y=\beta_{0}+\beta_{1}X_{1}+\beta_{2}X_{2}+\beta_{3}X_{3}+\beta_{12}X_{1}.X_{2}+\beta_{13}X_{1}.X_{3}+\beta_{23}X_{2}.X_{3}+\beta_{11}X^{2}+\beta_{22}X^{2}+\beta_{33}X^{2}$$

(Where *Y* is the predicted titer, β_0_ is the intercept, β_1,2,3_ represent the linear coefficients,

β_12,13,23_ represent the interaction coefficients, β_11,22,33_ are squared coefficients, and *X*_1_, *X*_2_*, X*_3_ are coded independent variables) [28].

#### 2.2.4 Final process optimization

Based on data from the iterative process (3.2), the optimal media composition was determined. However, it was hypothesized that a higher sucrose concentration might result in a higher titer if longer fermentation duration was allowed while keeping all other optimized parameters constant. Therefore, to fully optimize the media before testing scalability, it was necessary to understand if an increase in substrate and fermentation time exclusive of the other factors produced higher titers. To evaluate this, increased sucrose concentrations of 18 % (w/v) and 20 % (w/v) were tested. A higher inoculum volume of 22 % was also compared with the optimized 10 % for these increased concentrations of sucrose as it was assumed to be necessary with an increase in the substrate to avoid osmotic stress. The incubation period was increased from 4 days to 5 and 6 days. The other medium components were kept constant at their optimum concentrations: 0.0635 % (w/v) of YE, 0.0255 % (w/v) of (NH_4_)_2_SO_4_, 1.5 % (w/v) of K_2_HPO_4_, 0.02 % (w/v) of MgSO_4_, and 0.26 % (w/v) of NaCl. The initial pH was kept constant at the optimum 4.5.

#### 2.2.5 Characterization of pullulan by FTIR

The product was characterized using an ATR-FTIR spectrometer (Bruker Tensor 27). The spectral region measured was between 500–4000 cm^-1^. The dried sample was crushed to a powder in a mortar and pestle, and compared to a pullulan standard.

## 3 Results and discussion

### 3.1 OFAT

OFAT analysis showed that sucrose was the best carbon source producing 17.9 ± 0.8 g/L pullulan. A pH of 6 was optimum and 4 days was the optimum incubation period for production of pullulan. After OFAT a final titer of 21.0 ± 0.9 g/L was obtained. This was considered as the basis for the statistical optimization experiments. The results for the OFAT analysis are shown in Figs. B.1–B.3 (Appendix B).

### 3.2 Statistical optimization

#### 3.2.1 Trial 1

##### 3.2.1.1 PBD for media components

**Fig. 3:**
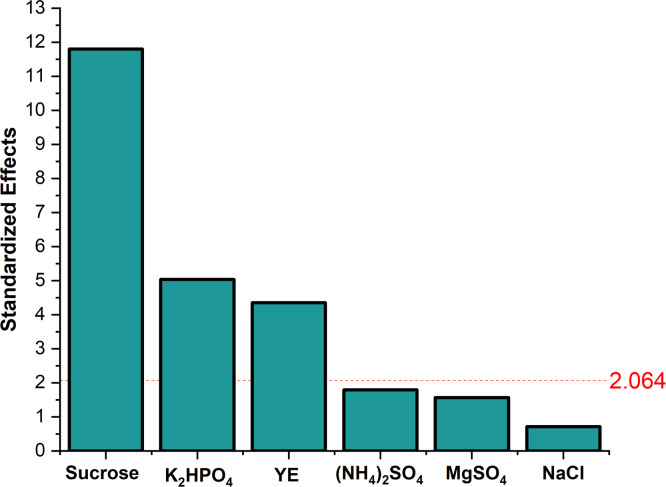
Pareto chart showing the significance of media components. The red line represents the line of significance, where the components above the line are significant components.

PBD was used to investigate the effect of six variables on EPS production. The design used and concentrations of the factor levels, along with the results are listed in Table A.1 (Appendix A) respectively. The Analysis of Variance (ANOVA) for the experiment design was calculated. Significant levels of each medium component were determined using the F-test. F-test indicated that sucrose, K_2_HPO_4_, and YE significantly affected pullulan production as seen in Table A.2 (Appendix A) and the pareto chart in Fig. 3. The pareto chart showed that sucrose had the highest significant effect followed by K_2_HPO_4_ and YE. The high significance of sucrose as a carbon source could be explained due to its role in promoting cell growth and determining pullulan titer as described by Singh et al. [29], where a genetically modified (GM) *A. pullulans* strain produced a highest titer of 60 g/L from 5 % sucrose. K_2_HPO_4_ regulates osmotic pressure, ionic strength, Na^+^/*K*^+^ ATPase, and supplies phosphorus to the organism, all of which have an impact on *A. pullulans* metabolism [30].

The main effect plot in Fig. B.4 (Appendix B) shows that product titer increases with a decrease in YE, and an increase in sucrose and K_2_HPO_4_, which is in line with the observations in Table A.2 (Appendix A). This is confirmed by previous works [5,31,32], where a signal for the pullulan formation was attributed to the depletion of nitrogen. The flask containing Sucrose 7 %, YE 0.05 %, (NH_4_)_2_SO_4_ 0.08 %, K_2_HPO_4_ 1 %, MgSO_4_ 0.03 %, and NaCl 0.02 % resulted in highest titer of 35.0 ± 0.5 g/L within the experiment.

The Multiple Correlation Coefficient (*R^2^*) was 0.88 which means that the model could explain 88 % of the runs conducted. This value was considered sufficient for screening for the significant factors since PBD uses only linear analysis and ignores all interactions. Adequate precision measures the signal-to-noise ratio where a ratio greater than 4 is desirable. The design’s ratio was 16.014 indicating an adequate signal. The model can therefore be used to navigate the design space.

The first-order polynomial model equation used for prediction was as follows:

(3) Y=16.21+7.41A−2.73B−1.13C+3.16D+0.98E+0.45F

Where *Y* represents the titer, *A* represents sucrose, *B* represents YE, *C* represents (NH_4_)_2_SO_4_, *D* represents K_2_HPO_4_, *E* represents MgSO_4_ and *F* represents NaCl.

##### 3.2.1.2 RSM for significant media components

RSM was done to explore interactions between the significant components. The design and concentrations are summarized in Table A.3 (Appendix A), respectively. Significant interactions were observed between the media components for pullulan formation during RSM as shown in Table A.4 (Appendix A). The evaluation of the design confirmed that the quadratic model is the best suited for this set of runs. Statistical evaluation was done using the p-value and F-value, where the overall significance of the regression model, fitness of the model terms, and the specific regression terms were analyzed. The smaller p-value and greater F-value show the statistical significance of the generated model.

**Fig. 4:**
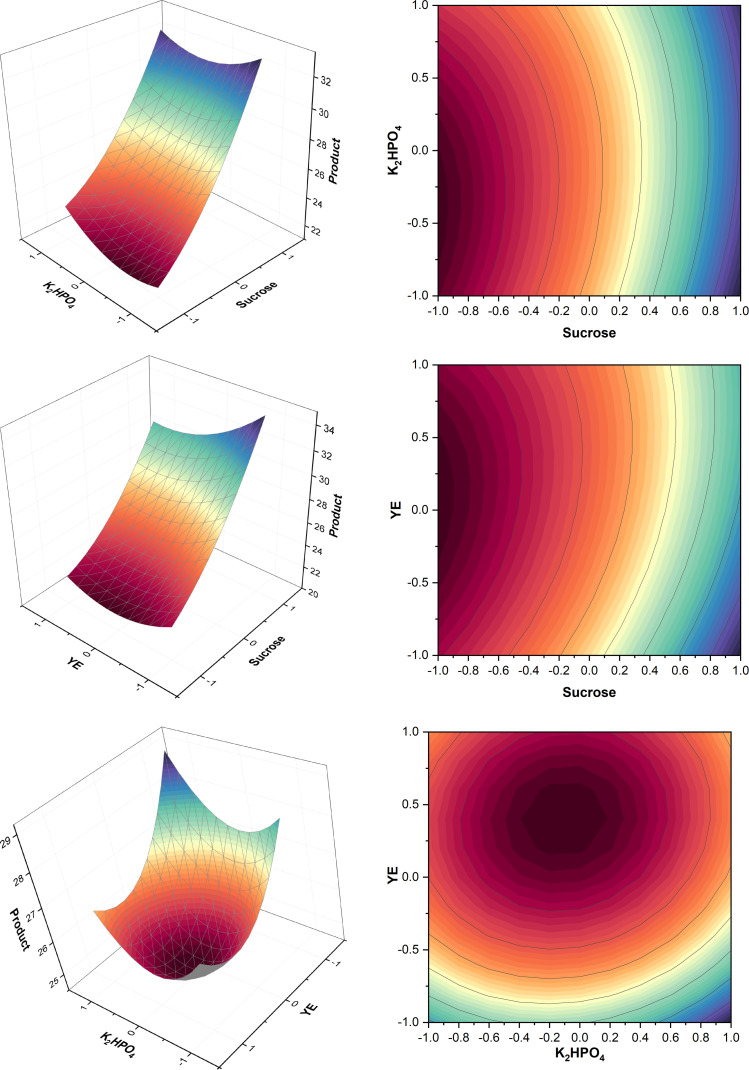
Surface and contour plots of product. K_2_HPO_4_, YE and sucrose were held at level zero, respectively (top to bottom).

ANOVA (Table A.4) showed that sucrose*sucrose, YE*YE, and K_2_HPO_4_* K_2_HPO_4_ interactions had the highest significance which meant there was no significant interaction between two different components of the media within this design. Fig. 4 demonstrates that the interaction between K_2_HPO_4_ and YE was close to negative, K_2_HPO_4_*sucrose and YE*sucrose did not have significant interaction. This could be because of a small range of variation between the component concentrations where the concentrations at which these interactions would be significant were not included in this design. However, the model F-value of 88.31 implies that the model is significant and only a 0.01 % chance indicates that an F-value this large could be explained due to noise. The run containing sucrose 8.682 % (w/v), K_2_HPO_4_ 1 % (w/v), and YE 0.016 % (w/v) showed the highest titer within the CCD giving 39 g/L of product. By the analysis of surface plots in Fig. 4 and comparing different titers from different runs, it was found that the higher sucrose and lower YE levels were better for the process as stated by West and Reed-Hamer [33]; i.e. sucrose 8.682 % (w/v), K_2_HPO_4_ 1 % (w/v), and YE 0.016 % (w/v) were optimum for higher pullulan titer. According to Göksungur et al. [34], pullulan production increases in response to an initial sugar concentration; after that, pullulan production is significantly reduced. Here, *A. pullulans* P56 produced a titer of 19.2 g/L after 111.8 h of incubation. This phenomenon was also confirmed by the work of Singh & Kaur [35] and Yoon et al. [36]. The absence of bell-shaped graphs reflected an opportunity for further optimization by increasing the media component concentrations and allowing more time for fermentation further tested in T2 (Section 3.2.2). 1 % K_2_HPO_4_ (w/v) was found to be the ideal concentration, which is higher than the concentration typically used in other studies [[37], [38], [39]]. This could be because though the metabolism remains the same, the optimum media composition remains strain specific [6]. Optimum lower YE level confirms results from the previous PBD (Section 3.2.1.1).

The validity of the design and the accuracy of the fit was proven by an *R^2^* value of 0.99. Generally, a regression model having an *R^2^* value greater than 0.9 is considered to have a very high correlation [40].

The regression model was obtained as follows:

(4) $$Y=25.20+5.17A+0.31B-1.07C+1.39A^{2}+1.12B^{2}+1.29C^{2}-0.56AB-0.81AC-0.19BC$$

Where *Y* represents the titer, *A* represents sucrose, *B* represents K_2_HPO_4_ and *C* represents YE. The titers and productivity are shown in Table A.3 (Appendix A).

##### 3.2.1.3 PBD for pH and incubation parameters

**Fig. 5:**
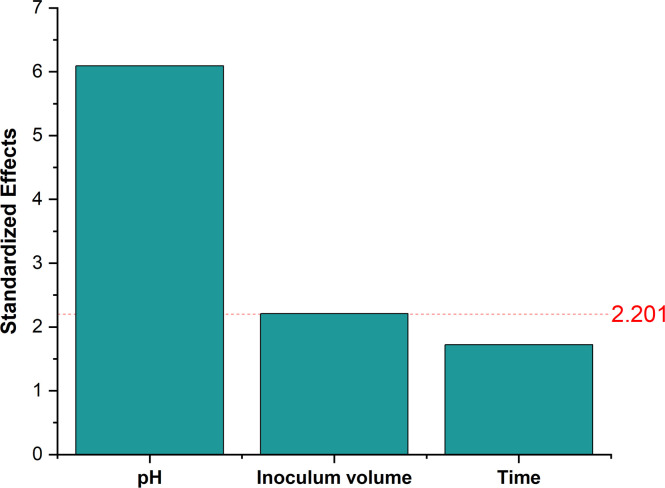
Pareto chart showing the significance of fermentation conditions.

This experiment followed the design depicted in Table A.5 (Appendix A). Design analysis showed that pH and inoculum volume were significant factors. Fig. B.5 (Appendix B) depicts the main effects, where a higher inoculum volume and a lower pH show higher EPS titers. This is in line with the research of Sharma et al. [41], where a lower pH was beneficial for pullulan production. Furthermore, higher inoculum volume would mean more cells utilizing the sugars simultaneously, producing more polymer. Fermentation time was found to be the least significant factor as seen in Tables A.5 and A.6 (Appendix A) and Fig. 5. 5 days of fermentation led to increased production. That might be due to a higher chance of complete consumption of substrates or passing by the late exponential phase where pullulan is produced which agrees with Jo et al. [42], who achieved 48.9 g/L pullulan from the strain *A. pullulans* MR. Run 11 conducted at pH 4, inoculum volume of 15 %, and fermentation time of 5 days showed the highest titer of 44.0 ± 0.5 g/L (Table A.5 (Appendix A)).

The equation used for prediction was as follows:

(5) Y=25.5+3.7G−10.4H+2.9I

*Y* represents the titer, *G* represents inoculum volume, *H* represents pH and *I* represents fermentation time. The experimental titer, predicted titer and productivity are shown in Table A.5 (Appendix A).

##### 3.2.1.4 RSM for pH and incubation parameters

**Fig. 6:**
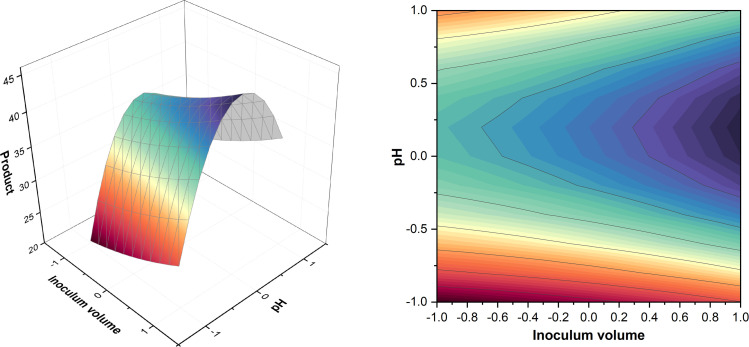
Surface and contour plots of product for interaction between pH and inoculum volume.

A summary of the design table and levels for the experiment is provided in Table A.7 (Appendix A). Based on ANOVA, Table A.8 (Appendix A) showed that the interaction between pH*pH has highest significance followed by pH*inoculum volume. This means that although pH has the highest effect on production, there is also some interaction between pH and inoculum volume as seen in Fig. 6, where level 0 for pH was optimum but the higher level of inoculum produced a slightly higher titer. Run 11 was conducted at pH 4.5 and inoculum volume of 22.07 % and showed the highest titer within the CCD giving 44.85 ± 1.51 g/L of product in agreement with Sharma et al. [41], who obtained the highest titer at the same pH. By the analysis of surface plots in Fig. 6 and comparing different titers from different runs, it was found that pH 4.78 and inoculum volume 22.07 % (v/v) were optimum for higher pullulan titer. Table A.7 (Appendix A) show the variations in the product under different conditions, again highlighting the need for further studies to confirm the effects. In the CCD, it was found that a pH higher than 6.5 or lower than 2.5 were not suitable for pullulan production which agrees with Sharma et al. [41] and Prasongsuk et al. [14]. These changes may also cause cell morphology alterations, influencing biosynthesis pathways [34].

Based on quadratic analysis, the regression model was obtained as follows:

(6) $$Y=40.75+4.59G+3.36H-13.39G^{2}+0.65H^{2}+0.28GH$$

Where *Y* represents the titer, *G* represents pH and *H* represents inoculum volume

#### 3.2.2 Trial 2

##### 3.2.2.1 RSM for increased ranges of media components

The 2nd Trial was done using optimum concentrations and conditions from T1, as a basis. Table A.9 (Appendix A) shows the design table and concentrations at different levels. As the analysis from T1 demonstrated the absence of bell curves and increase in product at higher concentrations of media components but no significant interactions between different components, a wider range seemed reasonable to test as a next step. ANOVA for the quadratic model in Table A.10 (Appendix A), showed that only sucrose was significantly affecting production while YE and K_2_HPO_4_ were insignificant although they were found to be significant in T1’s first PBD. This can be explained by the use of wider media components range compared to T1’s first CCD, i.e. YE’s 1.682 point here was 59 times greater than the −1.682 point, while in T1’s first CCD, it was only 5 times greater.

**Fig. 7:**
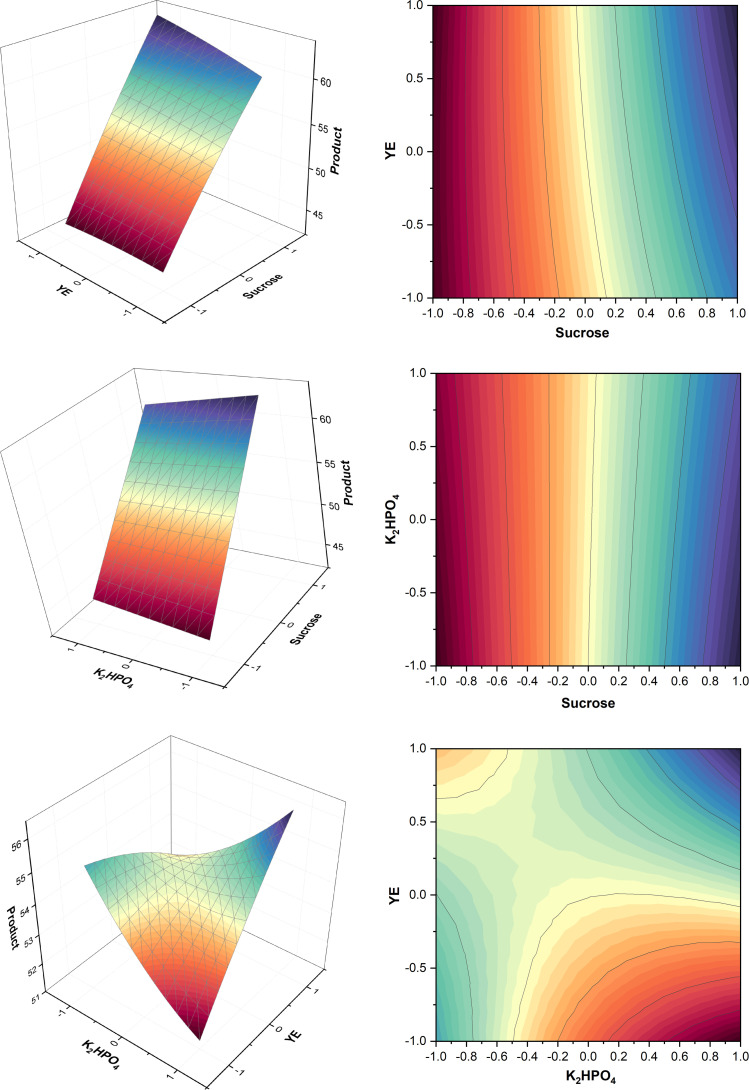
Surface and contour plots of product. K_2_HPO_4_, YE and sucrose were held at level zero, respectively (top to bottom).

Results are shown in Table A.9 (Appendix A) and Fig. 7. Run 16 containing sucrose 20.07 % (w/v), K_2_HPO_4_ 1.5 % (w/v), and YE 0.0635 % (w/v) showed the highest titer within the CCD giving 70.5 ± 1.5 g/L of product.

The surface plots in Fig. 7 showed that sucrose 20.07 % (w/v), K_2_HPO_4_ 1.5 % (w/v), and YE 0.0635 % (w/v) were optimum for higher pullulan titer. Although lower concentrations were optimum in T1, here the optimum concentrations for sucrose, K_2_HPO_4_, and YE were almost 2.3, 1.5, and 4 times higher, respectively. K_2_HPO_4_ 1.5 % (w/v) was again more than the typical optimum value in literature [[37], [38], [39]]. The increased values might be beneficial for higher biomass production and produce more polymer. The absence of bell-shaped surface plots could indicate that there were still substrates existing in the broth and therefore have further scope of optimization.

The quadratic model was obtained as follows:

(7) $$Y=53.85+9.00A-0.22B+0.85C-0.56A^{2}+0.41B^{2}-0.21C^{2}-0.94AB+0.94AC+1.69BC$$

Where *Y* represents the titer, *A* represents sucrose, *B* represents K_2_HPO_4_, *C* represents YE. Predicted titer and productivity were calculated and mentioned in Table A.9 (Appendix A).

##### 3.2.2.2 Iterated PBD for pH and incubation conditions

**Fig. 8:**
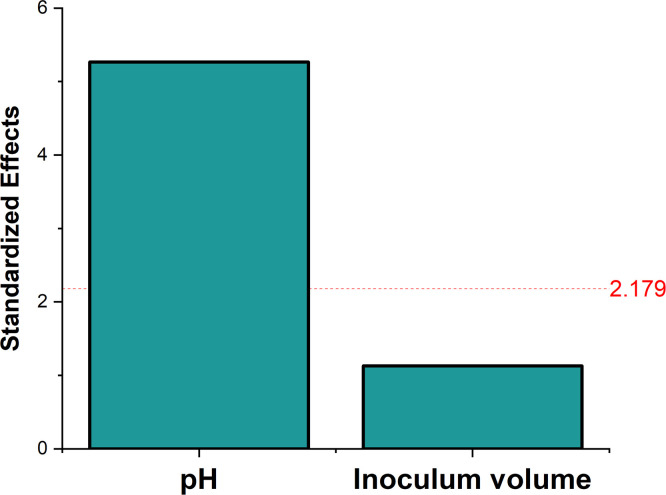
Pareto chart showing the significance of fermentation conditions.

**Fig. 9:**
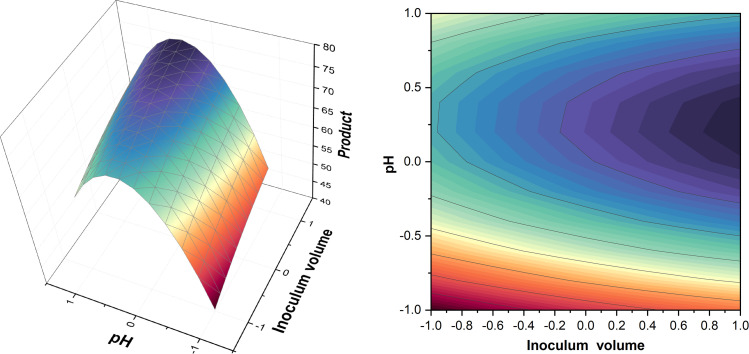
Surface and contour plots for product.

The experiment was conducted as per the design depicted in Table A.11 (Appendix A), describing the levels. Only pH and inoculum volume were included as factors as incubation period was not significant in T1 (Section 3.2.1.3). The design results shown in Table A.11, A.12 (Appendix A) and Fig. 8, Fig. 9 demonstrated similar results, where a lower pH and higher inoculum volume were better for production (Fig. B.6, Appendix B), when compared to PBD for incubation conditions from T1 (Section 3.2.1.3) even when the media components concentrations were increased based on results from Section 3.2.2.1. Fig. 8 showed a decrease in the significance of inoculum volume compared to previous PBDs which might be associated with the film formed, where cells sticking to the flasks’ inner wall can’t sufficiently contribute to the pullulan synthesis. This in turn led to inconsistent growth and substrate consumption, irrespective of the initially added volume concluding that the parameter was not significant as there was no interpretable correlation. Anderlei & Büchs [43] highlighted that uneven aeration and agitation in shake flask cultures can lead to heterogeneous conditions, impacting growth and metabolism.

The equation used for prediction was as follows:

(8) Y=39.5−17.7G+3.8H

Where *Y* represents the titer, *G* represents pH, *H* represents inoculum volume. The predicted titer and productivity were calculated and mentioned in Table A.11 (Appendix A).

##### 3.2.2.3 Iterated RSM for pH and incubation conditions

Table A.13 (Appendix A) shows the design and levels followed for the experiment. The design results presented in Tables A.13 and A.14 (Appendix A), as well as Fig. 9, showed some similar outcomes when compared to the CCD from T1 (Section 3.2.1.4). Optimum pH and inoculum volume obtained from surface plots were 4.84 and 22.07 %, respectively. pH value of 2.3 in run 12 wasn’t suitable for pullulan synthesis which agrees with results from T1 and Prasongsuk et al. [14], while pH of 6.62 in run 13 resulted in a titer of 58 ± 2 g/L unlike what was found in T1 and Prasongsuk et al. [14]. A decrease in inoculum volume significance was also seen in ANOVA shown in Table A.14 which coincided with the last PBD’s ANOVA shown in Table A.12. Runs (10–11) showed the importance of higher inoculum volume, where the use of higher inoculum volume gave higher titer. Run 11 conducted at pH 4.5 and inoculum volume of 22.07 % showed the highest titer giving 83.60 ± 1.95 g/L of product.

Quadratic model equation was:

(9) $$Y=74.77+9.35G+5.25H-17.79G^{2}-1.04H^{2}-0.85GH$$

Where *Y* represents the titer, *G* represents pH, *H* represents inoculum volume. Predicted titer and productivity are mentioned in Table A.13 (Appendix A).

#### 3.2.3 Trial 3

##### 3.2.3.1 PBD for integrated factors

**Fig. 10:**
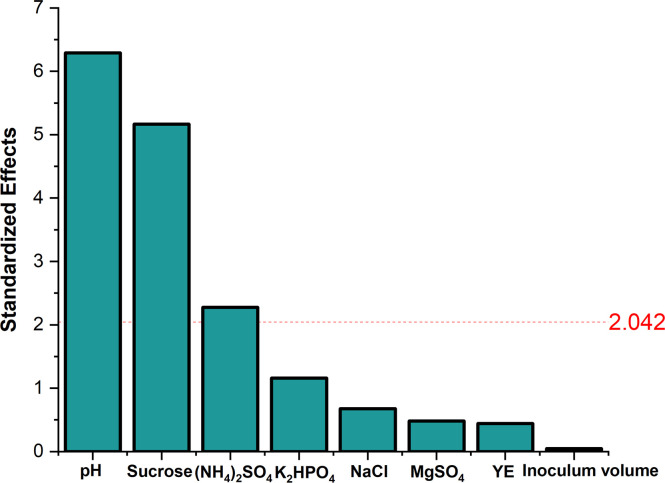
Pareto chart showing the significance of media components and fermentation conditions.

The design and concentrations of the experiment are presented in Table A.15 (Appendix A). ANOVA in Table A.16 (Appendix A) and pareto chart in Fig. 10 indicate that pH, sucrose, and (NH_4_)_2_SO_4_ significantly affected pullulan production. As pH was one of the factors integrated within the PBD along with the media components, the significance of components was expected to vary. It was found that pH had the highest significant effect followed by sucrose and (NH_4_)_2_SO_4_. As was found in previous designs, lower pH and (NH_4_)_2_SO_4_ and higher concentration of sucrose were increasing the production of pullulan as seen in Fig. B.7 (Appendix B). The significance of (NH_4_)_2_SO_4_ could be due to various reasons: (i) Its impact on pullulan-degrading enzyme activity and (ii) regulation of carbon flow according to Campbell et al. [44], and (iii) its high concentration could hinder the cell growth of *A. pullulans* and decrease EPS production, therefore depletion acts as a signal for EPS formation in *A. pullulans* fermentation [5,32,45]. When results were compared with the first PBD from T1, it was found that K_2_HPO_4_ lost its significance and was replaced by (NH_4_)_2_SO_4_ which might be explained by the possible interaction with pH where K_2_HPO_4_ could act as a buffer as stated by Stanbury et al. [46], and the small interaction between pH and (NH_4_)_2_SO_4_ which was found later in the next CCD experiment within T3. The insignificance of YE might be related to the simultaneous optimization of pH as seen previously.

Run 16 containing sucrose 20 % (w/v), YE 0.03 % (w/v), (NH_4_)_2_SO_4_ 0.04 % (w/v), K_2_HPO_4_ 1 % (w/v), MgSO_4_ 0.01 % (w/v), and NaCl 0.5 % (w/v) showed the highest titer of 78.0 ± 1.6 g/L.

First order polynomial equation was:

(10) Y=29.08+9.55A+2.14B−0.82C−4.21D+0.89E+1.25F−11.63G−0.09H

Where *Y* represents the titer, *A* represents sucrose, *B* represents K_2_HPO_4_, *C* represents YE, *D* represents (NH_4_)_2_SO_4_, *E* represents MgSO_4_, *F* represents NaCl, *G* represents pH and *H* represents inoculum volume. The predicted values of the product and productivity were calculated and are mentioned in Table A.15 (Appendix A).

##### 3.2.3.2 RSM for integrated factors

**Fig. 11:**
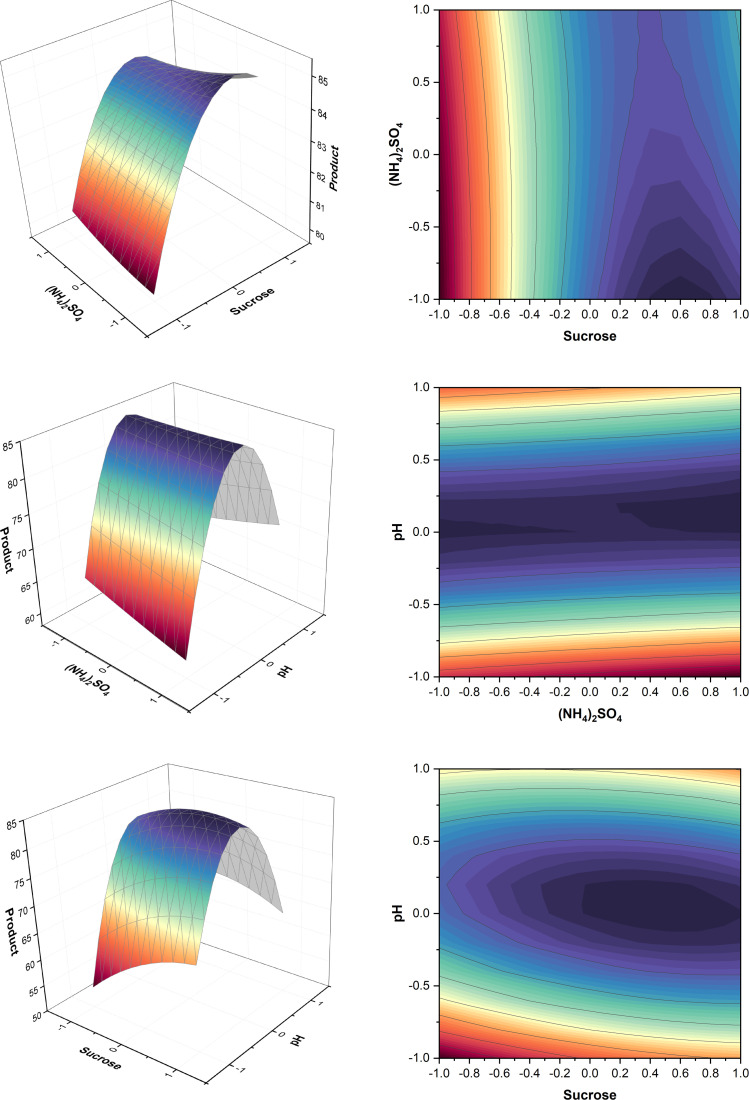
Surface and contour plots for product. pH, sucrose and (NH_4_)_2_SO_4_ were held at level zero, respectively (top to bottom).

The experimental design and levels depicted in Table A.17 (Appendix A) was used to conduct the experiment. Analysis was performed using ANOVA shown in Table A.18 (Appendix A). pH*pH and pH*sucrose interaction had the highest effect on the production. This can also be seen in Fig. 11, where the increase or decrease of (NH_4_)_2_SO_4_ did not alter how pH and sucrose affected production. Results are summarized in Table A.17 (Appendix A) show that run 12, which had 15 % (w/v) sucrose, a pH of 4.5, and 0.0255 % (w/v) (NH_4_)_2_SO_4_, resulted in the highest titer within the RSM, producing 88.7 ± 2.5 g/L of product.

Using surface plots in Fig. 11, it was found that, by changing sucrose concentration between runs, pullulan production increased from 6.59 % sucrose till the maximum at 18 % and then started to decrease again in agreement with literature [34–36]. The decrease could be attributed to the high sugar concentration in the medium which likely causes osmotic effects, such as decreased water activity and plasmolysis events, which result in a decrease in polysaccharide production [35].

The model’s equation was:

(11) $$Y=84.27+2.14A-3.48B-0.09C-2.16A^{2}-18.83B^{2}+0.15C^{2}-3.78AB-0.48AC+2.22BC$$

Where *Y* represents the titer, *A* represents sucrose, *B* represents pH and *C* represents (NH_4_)_2_SO_4_. Predicted titer and productivity are listed in Table A.17 (Appendix A).

### 3.3 Final process optimization

**Fig. 12:**
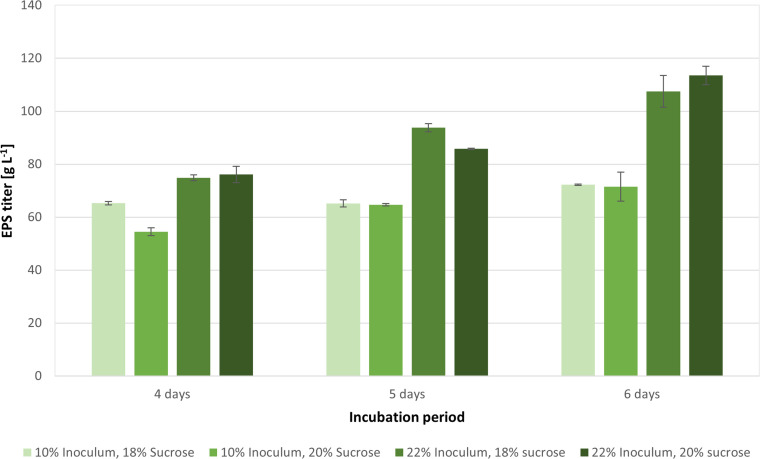
Plot showing EPS production over time for increased sucrose concentration and incubation periods.

Fig. 12 depicts the titers for the additional experiments based on the subsequent hypothesis (Section 2.2.4). 18 % (w/v) sucrose showed a faster production rate than 20 % (w/v), likely due to the slightly lower osmotic pressure at 18 % (w/v). However, between days 5 and 6, the production rate with 20 % (w/v) sucrose surpassed that of 18 % (w/v), suggesting that once the cells accommodated to the substrate consumption, the 2 % difference in sucrose concentration became less impactful.

With a 10 % inoculum volume and sucrose concentrations of 18 % (w/v) and 20 % (w/v), there was a marked difference in pullulan production by day 4. However, by day 6, the titers became comparable, suggesting that the slight difference in sucrose concentration impacts production more at lower inoculum volumes. This is comparable with the work of Cheng et al. [7], where it was described that larger inoculum sizes led to accelerated substrate consumption and higher titers of pullulan, as proven also in other studies [41,47]. A higher inoculum volume could therefore be attributed to a reduced lag phase, leading to a faster start of biomass formation and higher pullulan production. The optimized media, with 20 % (w/v) sucrose, 22 % inoculum volume, and a 6-day fermentation time resulted in 113.5 ± 3.5 g/L of pullulan production, the highest titer of crude pullulan ever reported for a wild-type *A. pullulans* (ATCC 42023).

### 3.4 Characterization of pullulan by FTIR

**Fig. 13:**
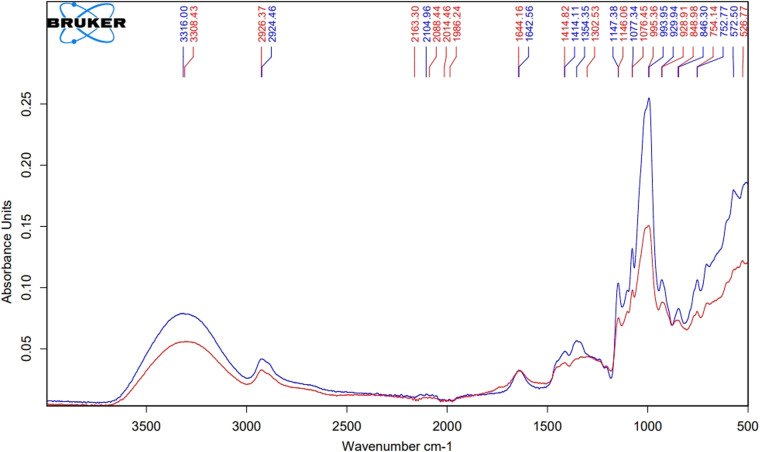
FTIR spectrum of dried pullulan sample (Red) vs pullulan standard (Blue).

The FTIR spectra for dried pullulan produced by *A. pullulans* was compared with standard pullulan (Fig. 13), showing very similar signals. O-H stretching was observed at 3308.43 for the sample and 3316 for the standard. Peaks at 2926.37 and 2924.46 depicted C-H stretching respectively. Absorption for O-CO stretching was seen at 1644.16 and 1642.56 respectively. C-O–H bending was observed for the pullulan sample at 1302.53 and 1354.35 for standard pullulan. Absorption peaks at 1146.06 and 1147.38 showed C-O–C stretching respectively. C-O stretching was depicted at 995.36 and 993.95 respectively for the sample and standard. Characteristic α-configuration was attributed to bands at 848.98 for pullulan sample and 846.30 for the standard [48].

## 4 Conclusion

High pullulan titers could be achieved by optimization of media components and fermentation conditions. OFAT showed that sucrose produced more EPS when compared to glucose and fructose. The production medium developed, containing 5 % (w/v) sucrose and 0.1 % (w/v) YE produced a maximum EPS titer of 21 ± 0.94 g/L at pH 6. OFAT also concluded that the raw sources (cane molasses and glycerol) might need pre-treatments to be used as productive carbon sources. On day 4, EPS production was maximum, and visible changes like a shift in the culture colour and broth thickening, could be observed over time. To further statistically optimize the media components and fermentation conditions for pullulan production in shake flasks through different iterative trials, DBTL cycle was applied from one trial to another. The starting points were based on the results from OFAT. In T1, it was discovered that sucrose, YE, K_2_HPO_4_, pH, and inoculum volume significantly influenced pullulan production. However, in T2, inoculum volume was determined to be insignificant due to the sticking of cells to the wall leading to inconsistent substrate consumption. T3 indicated that only pH and sucrose significantly affected production. In T3, for the first time, both media components and fermentation conditions were combined to be optimized simultaneously. This resulted in the highest pullulan titer of 113.5 ± 3.5 g/L for a wild type strain when compared to Kim et al. [49] who obtained 54 g/L using the exact same strain.

The strain showed tolerance to hyperosmotic stress, producing high titers of pullulan even with high sucrose concentration in T2 and T3. Therefore, *A. pullulans* (DSM 3042) proves to have great potential and may emerge as a cost-effective candidate for large-scale production. For further optimization future investigations could be performed such as quantifying substrate and product kinetics to design fed-batch fermentations scaled up in reactors, aiming for high pullulan productivity with minimum biomass growth. Biomass assessment could be done to confirm the productive morphological phase by understanding the growth pattern and production at different growth stages. In-situ product removal and cell-recycling might be interesting aspects when considering high viscosities of the broth. Optimization of reactor parameters to even further increase titers could be investigated, making the overall process cost-effective for industrially produced biopolymers.

## CRediT authorship contribution statement

**Venessa Dsouza:** Writing – original draft, Visualization, Validation, Project administration, Methodology, Formal analysis, Conceptualization. **Goldee Thoidingjam:** Investigation. **Abdelrahman Saleh:** Investigation, Formal analysis. **Michael Zavrel:** Writing – review & editing, Supervision.

## Declaration of competing interest

The authors declare that they have no known competing financial interests or personal relationships that could have appeared to influence the work reported in this paper.

## Data Availability

Data will be made available on request.

## Appendix Supplementary materials

Image, application 1
