# Supplementary material for: An iterative approach to statistical optimization of exopolysaccharide produced by fermentation of Aureobasidium pullulans
Source: Biotechnol Rep (Amst). 2025 Aug 15;47:e00914. doi: 10.1016/j.btre.2025.e00914 (PMC12414291; doi:10.1016/j.btre.2025.e00914)
Supplement: Supplementary file 1 [file mmc1.docx]

Appendix A

Table A.1: PBD matrix for levels of media components in T1

| **RUN** | **Sucrose** | **YE** | | **(NH_4_)_2_SO_4_** | **K_2_HPO_4_** | | **MgSO_4_** | **NaCl** | | **Titer (g/L)** | **Predicted titer (g/L)** |
| --- | --- | --- | --- | --- | --- | --- | --- | --- | --- | --- | --- |
| 1 | 0 | 0 | | 0 | 0 | | 0 | 0 | | 19.5 | 16.2 |
| 2 | 1 | 1 | | -1 | -1 | | 1 | 1 | | 21.5 | 20.3 |
| 3 | -1 | -1 | | 1 | -1 | | -1 | 1 | | 6.5 | 6.7 |
| 4 | -1 | -1 | | -1 | 1 | | -1 | 1 | | 11.0 | 15.3 |
| 5 | 1 | 1 | | 1 | -1 | | -1 | -1 | | 14.5 | 15.2 |
| 6 | 1 | -1 | | 1 | 1 | | 1 | 1 | | 32.5 | 29.8 |
| 7 | 1 | 1 | | -1 | 1 | | 1 | 1 | | 26.0 | 26.6 |
| 8 | -1 | 1 | | 1 | 1 | | 1 | -1 | | 7.0 | 8.6 |
| 9 | -1 | -1 | | -1 | -1 | | -1 | -1 | | 10.0 | 8.1 |
| 10 | -1 | 1 | | 1 | -1 | | 1 | 1 | | 6.0 | 3.2 |
| 11 | -1 | 1 | | -1 | -1 | | -1 | 1 | | 7.5 | 3.5 |
| 12 | -1 | 1 | | -1 | 1 | | -1 | -1 | | 6.5 | 8.9 |
| 13 | 1 | 1 | | -1 | 1 | | -1 | 1 | | 24.0 | 24.6 |
| 14 | -1 | -1 | | -1 | -1 | | 1 | 1 | | 11.0 | 10.9 |
| 15 | 1 | -1 | | -1 | -1 | | 1 | -1 | | 23.5 | 24.9 |
| 16 | 0 | 0 | | 0 | 0 | | 0 | 0 | | 19.0 | 16.2 |
| 17 | 1 | 1 | | -1 | 1 | | 1 | -1 | | 24.5 | 25.7 |
| 18 | -1 | 1 | | -1 | -1 | | 1 | -1 | | 7.5 | 4.6 |
| 19 | 1 | 1 | | 1 | -1 | | -1 | -1 | | 9.5 | 15.2 |
| 20 | -1 | -1 | | -1 | 1 | | 1 | -1 | | 12.0 | 16.4 |
| 21 | -1 | -1 | | 1 | -1 | | 1 | -1 | | 8.5 | 7.8 |
| 22 | 1 | -1 | | 1 | -1 | | 1 | 1 | | 15.0 | 23.5 |
| 23 | -1 | -1 | | 1 | 1 | | -1 | -1 | | 11.0 | 12.1 |
| 24 | 1 | -1 | | 1 | 1 | | -1 | 1 | | 31.5 | 27.9 |
| 25 | -1 | 1 | | 1 | 1 | | -1 | 1 | | 8.0 | 7.6 |
| 26 | 1 | -1 | | 1 | 1 | | 1 | -1 | | 35.0 | 28.9 |
| 27 | 1 | -1 | | -1 | -1 | | -1 | 1 | | 22.0 | 23.8 |
| 28 | 1 | -1 | | -1 | 1 | | -1 | -1 | | 31.5 | 29.2 |
| 29 | -1 | 1 | | 1 | 1 | | 1 | 1 | | 6.5 | 9.5 |
| 30 | 1 | 1 | | 1 | -1 | | -1 | -1 | | 15.5 | 15.2 |
| 31 | 0 | 0 | | 0 | 0 | | 0 | 0 | | 18.5 | 16.2 |
|  |  |  | |  |  | |  |  | |  |  |
| **Factors** | | | **Levels** | | | | | | | | |
|  | | | Low (-1) | | | Center (0) | | | High (1) | | |
| Sucrose | | | 3 %(w/v) | | | 5 %(w/v) | | | 7 %(w/v) | | |
| YE | | | 0.05 %(w/v) | | | 0.1 %(w/v) | | | 0.2 %(w/v) | | |
| (NH_4_)_2_SO_4_ | | | 0.04 %(w/v) | | | 0.06 %(w/v) | | | 0.08 %(w/v) | | |
| K_2_HPO_4_ | | | 0.1 %(w/v) | | | 0.5 %(w/v) | | | 1 %(w/v) | | |
| MgSO_4_ | | | 0.01 %(w/v) | | | 0.02 %(w/v) | | | 0.03 %(w/v) | | |
| NaCl | | | 0.02 %(w/v) | | | 0.1 %(w/v) | | | 0.5 %(w/v) | | |
|  | | |  | | |  | | |  | | |
| **Constant parameters** | | | | | | | | | | | |
| **Parameter** | | | **Constant value** | | |  | | |  | | |
| pH | | | 6 | | |  | | |  | | |
| Inoculum volume | | | 10 % | | |  | | |  | | |
| Fermentation time | | | 4 days | | |  | | |  | | |

Table A.2: ANOVA for linear model for PBD for media components in T1

|  | **DF** | **Sum of Squares** | **Mean Square** | **F Value** | **Prob>F** |
| --- | --- | --- | --- | --- | --- |
| Sucrose | 1 | 1537.7 | 1537.7 | 125.8 | 5.00E-11** |
| YE | 1 | 209.0 | 209.0 | 17.1 | 3.75E-4** |
| (NH_4_)_2_SO_4_ | 1 | 35.4 | 35.4 | 2.9 | 0.10 |
| K_2_HPO_4_ | 1 | 279.7 | 279.7 | 22.9 | 7.20E-5** |
| MgSO_4_ | 1 | 27.0 | 27.0 | 2.2 | 0.15 |
| NaCl | 1 | 5.6 | 5.6 | 0.5 | 0.51 |
| Error | 24 | 293.4 | 12.2 |  |  |
| Total | 30 | 2387.9 |  |  |  |

^** Statistically significant at 99% of probability level^

Table A.3: RSM design matrix for levels of significant media components in T1

| **Run** | **Sucrose** | | **K_2_HPO_4_** | **YE** | | **Titer (g/L)** | | **Predicted titer (g/L)** | |
| --- | --- | --- | --- | --- | --- | --- | --- | --- | --- |
| 1 | -1 | | -1 | -1 | | 23.0 | | 23.0 | |
| 2 | 1 | | -1 | -1 | | 35.5 | | 36.1 | |
| 3 | -1 | | 1 | -1 | | 25.0 | | 25.1 | |
| 4 | 1 | | 1 | -1 | | 35.5 | | 36.0 | |
| 5 | -1 | | -1 | 1 | | 22.5 | | 22.9 | |
| 6 | 1 | | -1 | 1 | | 32.0 | | 32.7 | |
| 7 | -1 | | 1 | 1 | | 24.0 | | 24.3 | |
| 8 | 1 | | 1 | 1 | | 31.0 | | 31.9 | |
| 9 | 0 | | 0 | 0 | | 25.5 | | 25.2 | |
| 10 | 0 | | 0 | 0 | | 25.0 | | 25.2 | |
| 11 | 0 | | 0 | 0 | | 25.0 | | 25.2 | |
| 12 | 0 | | 0 | 0 | | 25.0 | | 25.2 | |
| 13 | 0 | | 0 | 0 | | 25.5 | | 25.2 | |
| 14 | 0 | | 0 | 0 | | 25.0 | | 25.2 | |
| 15 | -1.682 | | 0 | 0 | | 20.5 | | 20.4 | |
| 16 | 1.682 | | 0 | 0 | | 39.0 | | 37.8 | |
| 17 | 0 | | -1.682 | 0 | | 28.5 | | 27.9 | |
| 18 | 0 | | 1.682 | 0 | | 29.5 | | 28.9 | |
| 19 | 0 | | 0 | -1.682 | | 31.0 | | 30.7 | |
| 20 | 0 | | 0 | 1.682 | | 28.0 | | 27.1 | |
|  |  | |  |  | |  | |  |  |
| **Levels** | | **Sucrose** | | | **K_2_HPO_4_** | | **YE** | | |
| 1.682 | | 8.682 %(w/v) | | | 1.1682 %(w/v) | | 0.08364 %(w/v) | | |
| 1 | | 8 %(w/v) | | | 1.1 %(w/v) | | 0.07 %(w/v) | | |
| 0 | | 7 %(w/v) | | | 1 %(w/v) | | 0.05 %(w/v) | | |
| -1 | | 6 %(w/v) | | | 0.9 %(w/v) | | 0.03 %(w/v) | | |
| -1.682 | | 5.318 %(w/v) | | | 0.8318 %(w/v) | | 0.01636 %(w/v) | | |
|  | |  | | |  | |  | | |
| **Constant parameters** | | | | | | | | | |
| **Parameter** | | **Constant value** | | |  | |  | | |
| (NH_4_)_2_SO_4_ | | 0.04 %(w/v) | | |  | |  | | |
| MgSO_4_ | | 0.03 %(w/v) | | |  | |  | | |
| NaCl | | 0.5 %(w/v) | | |  | |  | | |
| pH | | 6 | | |  | |  | | |
| Inoculum volume | | 10 % | | |  | |  | | |
| Incubation time | | 4 days | | |  | |  | | |

Table A.4: ANOVA for quadratic model for RSM of significant media components in T1

|  | **DF** | **Sum of Squares** | **Mean Square** | **F Value** | **Prob>F** |
| --- | --- | --- | --- | --- | --- |
| Sucrose | 1 | 365.1 | 365.1 | 646.9 | 2.03E-10** |
| K_2_HPO_4_ | 1 | 1.3 | 1.3 | 2.3 | 0.16 |
| YE | 1 | 15.5 | 15.5 | 27.5 | 3.79E-4** |
| Sucrose.Sucrose | 1 | 20.0 | 20.0 | 35.4 | 1.41E-4** |
| K_2_HPO_4_. K_2_HPO_4_ | 1 | 14.3 | 14.3 | 25.4 | 5.09E-4** |
| YE.YE | 1 | 24.3 | 24.3 | 43.0 | 6.42E-5** |
| Sucrose.K_2_HPO_4_ | 1 | 2.5 | 2.5 | 4.5 | 0.06 |
| Sucrose.YE | 1 | 5.3 | 5.3 | 9.4 | 0.01* |
| K_2_HPO_4_.YE | 1 | 0.3 | 0.3 | 0.5 | 0.50 |
| Error | 10 | 5.6 | 0.6 |  |  |
| Lack of fit | 5 | 5.3 | 1.1 | 15.9 | 4.32E-3** |
| Pure Error | 5 | 0.3 | 0.1 |  |  |
| Total | 19 | 454.2 |  |  |  |

^* Statistically significant at 95% of probability level ** Statistically significant at 99% of probability level^

Table A.5: PBD matrix for levels of pH and incubation parameters in T1

| **Run** | **Inoculum volume** | | **pH** | **Time** | | **Titer (g/L)** | **Predicted titer (g/L)** |
| --- | --- | --- | --- | --- | --- | --- | --- |
| 1 | 1 | | 1 | -1 | | 14.0 | 15.9 |
| 2 | -1 | | 1 | 1 | | 15.0 | 14.2 |
| 3 | 1 | | -1 | 1 | | 41.0 | 42.7 |
| 4 | -1 | | 1 | -1 | | 8.5 | 8.3 |
| 5 | -1 | | -1 | 1 | | 31.5 | 35.1 |
| 6 | -1 | | -1 | -1 | | 26.5 | 29.2 |
| 7 | 1 | | -1 | -1 | | 31.5 | 36.8 |
| 8 | 1 | | 1 | -1 | | 13.5 | 15.9 |
| 9 | 1 | | 1 | 1 | | 16.0 | 21.8 |
| 10 | -1 | | 1 | 1 | | 7.5 | 14.2 |
| 11 | 1 | | -1 | 1 | | 44.0 | 42.7 |
| 12 | -1 | | -1 | -1 | | 25.5 | 29.2 |
| 13 | 0 | | 0 | 0 | | 35.5 | 25.5 |
| 14 | 0 | | 0 | 0 | | 36.0 | 25.5 |
| 15 | 0 | | 0 | 0 | | 36.5 | 25.5 |
|  |  | |  |  | |  |  |
| **Factors** | | **Levels** | | | | | |
|  | | Low (-1) | | | Center (0) | | High (1) |
| Inoculum volume | | 5 % | | | 10 % | | 15 % |
| pH | | 4 | | | 6 | | 8 |
| Fermentation time | | 3 days | | | 4 days | | 5 days |
|  | |  | | |  | |  |
| **Constant parameters** | | | | | | | |
| **Parameter** | | **Constant value** | | |  | |  |
| Sucrose | | 8.682 %(w/v) | | |  | |  |
| YE | | 0.016 %(w/v) | | |  | |  |
| (NH_4_)_2_SO_4_ | | 0.04 %(w/v) | | |  | |  |
| K_2_HPO_4_ | | 1 %(w/v) | | |  | |  |
| MgSO_4_ | | 0.03 %(w/v) | | |  | |  |
| NaCl | | 0.5 %(w/v) | | |  | |  |

Table A.6: ANOVA for linear model of PBD for pH and incubation parameters in T1

|  | **DF** | **Sum of Squares** | **Mean Square** | **F Value** | **Prob>F** |
| --- | --- | --- | --- | --- | --- |
| Inoculum volume | 1 | 172.5 | 172.5 | 3.9 | 0.07 |
| pH | 1 | 1312.5 | 1312.5 | 29.7 | 2.02E-4** |
| Time | 1 | 105.0 | 105.0 | 2.4 | 0.15 |
| Error | 11 | 486.4 | 44.2 |  |  |
| Total | 14 | 2076.5 |  |  |  |

^** Statistically significant at 99% of probability level^

Table A.7: RSM design matrix for levels of pH and incubation parameters in T1

| **Run** | **pH** | | **Inoculum volume** | | | **Product (g/L)** | | **Predicted titer (g/L)** |
| --- | --- | --- | --- | --- | --- | --- | --- | --- |
| 1 | -1 | | -1 | | | 23.4 | | 20.3 |
| 2 | -1 | | 1 | | | 28.0 | | 26.5 |
| 3 | 1 | | -1 | | | 33.5 | | 29.0 |
| 4 | 1 | | 1 | | | 39.3 | | 36.3 |
| 5 | 0 | | 0 | | | 42.5 | | 40.8 |
| 6 | 0 | | 0 | | | 39.9 | | 40.8 |
| 7 | 0 | | 0 | | | 41.7 | | 40.8 |
| 8 | 0 | | 0 | | | 41.5 | | 40.8 |
| 9 | 0 | | 0 | | | 38.3 | | 40.8 |
| 10 | 0 | | -1.414 | | | 33.3 | | 37.3 |
| 11 | 0 | | 1.414 | | | 44.9 | | 46.8 |
| 12 | -1.414 | | 0 | | | 5.5 | | 7.5 |
| 13 | 1.414 | | 0 | | | 16.4 | | 20.5 |
|  |  | |  | | |  | |  |
| **Levels** | | **pH** | | | **Inoculum volume** | |  |  |
| 1.414 | | 6.62 | | | 22.07 % | |  |  |
| 1 | | 6 | | | 20 % | |  |  |
| 0 | | 4.5 | | | 15 % | |  |  |
| -1 | | 3 | | | 10 % | |  |  |
| -1.414 | | 2.3 | | | 7.93 % | |  |  |
|  | |  | | |  | |  |  |
| **Constant parameters** | | | | | | |  |  |
| **Parameter** | | **Constant value** | |  | | |  |  |
| Sucrose | | 8.682 %(w/v) | | |  | |  |  |
| YE | | 0.016 %(w/v) | | |  | |  |  |
| (NH_4_)_2_SO_4_ | | 0.04 %(w/v) | | |  | |  |  |
| K_2_HPO_4_ | | 1 %(w/v) | | |  | |  |  |
| MgSO_4_ | | 0.03 %(w/v) | | |  | |  |  |
| NaCl | | 0.5 %(w/v) | | |  | |  |  |
| Incubation time | | 4 days | | |  | |  |  |

Table A.8: ANOVA for quadratic model for RSM for pH and incubation parameters in T1

|  | **DF** | **Sum of Squares** | **Mean Square** | **F Value** | **Prob>F** |
| --- | --- | --- | --- | --- | --- |
| pH | 1 | 169.0 | 169.0 | 12.8 | 0.01* |
| Inoculum volume | 1 | 90.2 | 90.2 | 6.8 | 0.04* |
| pH.pH | 1 | 1286.2 | 1286.2 | 97.2 | 1.00E-5** |
| Inoculum volume.Inoculum volume | 1 | 3.0 | 3.0 | 0.2 | 0.65 |
| pH.Inoculum volume | 1 | 0.3 | 0.3 | 0.0 | 0.88 |
| Error | 7 | 92.6 | 13.2 |  |  |
| Lack of fit | 3 | 81.2 | 27.1 | 9.5 | 0.03* |
| Pure Error | 4 | 11.4 | 2.9 |  |  |
| Total | 12 | 1641.3 |  |  |  |

^* Statistically significant at 95% of probability level ** Statistically significant at 99% of probability level^

Table A.9: RSM design matrix for levels of increased ranges of media components in T2

| **Run** | **Sucrose** | | **K_2_HPO_4_** | | **YE** | **Titer (g/L)** | | **Predicted titer (g/L)** |
| --- | --- | --- | --- | --- | --- | --- | --- | --- |
| 1 | -1 | | -1 | | -1 | 47.0 | | 45.5 |
| 2 | 1 | | -1 | | -1 | 64.0 | | 63.6 |
| 3 | -1 | | 1 | | -1 | 45.5 | | 43.6 |
| 4 | 1 | | 1 | | -1 | 56.0 | | 57.9 |
| 5 | -1 | | -1 | | 1 | 43.5 | | 42.0 |
| 6 | 1 | | -1 | | 1 | 61.5 | | 63.8 |
| 7 | -1 | | 1 | | 1 | 46.0 | | 46.8 |
| 8 | 1 | | 1 | | 1 | 63.0 | | 64.8 |
| 9 | 0 | | 0 | | 0 | 54.5 | | 53.9 |
| 10 | 0 | | 0 | | 0 | 56.5 | | 53.9 |
| 11 | 0 | | 0 | | 0 | 51.5 | | 53.9 |
| 12 | 0 | | 0 | | 0 | 54.0 | | 53.9 |
| 13 | 0 | | 0 | | 0 | 53.0 | | 53.9 |
| 14 | 0 | | 0 | | 0 | 53.5 | | 53.9 |
| 15 | -1.682 | | 0 | | 0 | 34.5 | | 37.1 |
| 16 | 1.682 | | 0 | | 0 | 70.5 | | 67.4 |
| 17 | 0 | | -1.682 | | 0 | 54.5 | | 55.4 |
| 18 | 0 | | 1.682 | | 0 | 56.0 | | 54.6 |
| 19 | 0 | | 0 | | -1.682 | 50.5 | | 51.8 |
| 20 | 0 | | 0 | | 1.682 | 56.5 | | 54.7 |
|  |  | |  | |  |  | |  |
| **Levels** | | **Sucrose** | | **K_2_HPO_4_** | | | **YE** | |
| 1.682 | | 20.069 %(w/v) | | 2.341 %(w/v) | | | 0.124893 %(w/v) | |
| 1 | | 17 %(w/v) | | 2 %(w/v) | | | 0.1 %(w/v) | |
| 0 | | 12.5 %(w/v) | | 1.5 %(w/v) | | | 0.0635 %(w/v) | |
| -1 | | 8 %(w/v) | | 1 %(w/v) | | | 0.027 %(w/v) | |
| -1.682 | | 4.931 %(w/v) | | 0.659 %(w/v) | | | 0.002107 %(w/v) | |
|  | |  | |  | | |  | |
| **Constant parameters** | | | | | | | | |
| **Parameter** | | **Constant value** | |  | | |  | |
| (NH_4_)_2_SO_4_ | | 0.06 %(w/v) | |  | | |  | |
| MgSO_4_ | | 0.02 %(w/v) | |  | | |  | |
| NaCl | | 0.1 %(w/v) | |  | | |  | |
| pH | | 4.78 | |  | | |  | |
| Inoculum volume | | 22.07 % | |  | | |  | |
| Incubation time | | 4 days | |  | | |  | |

Table A.10: ANOVA for quadratic model for RSM for increased ranges of media components in T2

|  | **DF** | **Sum of Squares** | **Mean Square** | **F Value** | **Prob>F** |
| --- | --- | --- | --- | --- | --- |
| Sucrose | 1 | 1108.6 | 1108.6 | 189.3 | 2.04E-6** |
| K_2_HPO_4_ | 1 | 0.7 | 0.7 | 0.1 | 0.75 |
| YE | 1 | 9.8 | 9.8 | 1.7 | 0.22 |
| Sucrose.Sucrose | 1 | 4.9 | 4.9 | 0.8 | 0.38 |
| K_2_HPO_4_.K_2_HPO_4_ | 1 | 2.7 | 2.7 | 0.5 | 0.51 |
| YE.YE | 1 | 0.6 | 0.6 | 0.1 | 0.75 |
| Sucrose.K_2_HPO_4_ | 1 | 7.0 | 7.0 | 1.2 | 0.30 |
| Sucrose.YE | 1 | 7.0 | 7.0 | 1.2 | 0.30 |
| K_2_HPO_4_.YE | 1 | 22.8 | 22.8 | 3.9 | 0.08 |
| Error | 10 | 58.6 | 5.9 |  |  |
| Lack of fit | 5 | 44.7 | 9.0 | 3.2 | 0.11 |
| Pure Error | 5 | 13.8 | 2.8 |  |  |
| Total | 19 | 1222.8 |  |  |  |

^** Statistically significant at 99% of probability level^

Table A.11: Iterated PBD matrix for levels of pH and incubation conditions in T2

| **Run** | **Inoculum volume** | | **pH** | **Titer (g/L)** | | **Predicted titer (g/L)** | |
| --- | --- | --- | --- | --- | --- | --- | --- |
| 1 | 1 | | 1 | 7.1 | | 25.6 | |
| 2 | 1 | | -1 | 54.5 | | 61.1 | |
| 3 | -1 | | 1 | 31.3 | | 18.0 | |
| 4 | 1 | | -1 | 59.0 | | 61.1 | |
| 5 | -1 | | -1 | 44.9 | | 53.4 | |
| 6 | -1 | | -1 | 50.6 | | 53.4 | |
| 7 | -1 | | 1 | 4.4 | | 18.0 | |
| 8 | 1 | | 1 | 23.2 | | 25.6 | |
| 9 | 1 | | 1 | 31.4 | | 25.6 | |
| 10 | 1 | | -1 | 57.5 | | 61.1 | |
| 11 | -1 | | 1 | 6.0 | | 18.0 | |
| 12 | -1 | | -1 | 49.8 | | 53.4 | |
| 13 | 0 | | 0 | 56.0 | | 39.5 | |
| 14 | 0 | | 0 | 59.5 | | 39.5 | |
| 15 | 0 | | 0 | 57.7 | | 39.5 | |
|  |  | |  |  | |  | |
| **Factors** | | **Levels** | | | | | |
|  | | Low (-1) | | | Center (0) | | High (1) |
| Inoculum volume | | 5 % | | | 10 % | | 15 % |
| pH | | 4 | | | 6 | | 8 |
|  | |  | | |  | |  |
| **Constant parameters** | | | | | | | |
| **Parameter** | | **Constant value** | | |  | |  |
| Sucrose | | 20.07 %(w/v) | | |  | |  |
| YE | | 0.0635 %(w/v) | | |  | |  |
| (NH_4_)_2_SO_4_ | | 0.06 %(w/v) | | |  | |  |
| K_2_HPO_4_ | | 1.5 %(w/v) | | |  | |  |
| MgSO_4_ | | 0.02 %(w/v) | | |  | |  |
| NaCl | | 0.1 %(w/v) | | |  | |  |
| Incubation time | | 4 days | | |  | |  |

Table A.12: ANOVA for linear model for iterated PBD for pH and incubation conditions in T2

|  | **DF** | **Sum of Squares** | **Mean Square** | **F Value** | **Prob>F** |
| --- | --- | --- | --- | --- | --- |
| Inoculum volume | 1 | 174.2 | 174.2 | 1.0 | 0.33 |
| pH | 1 | 3776.7 | 3776.7 | 22.2 | 1.00E-3** |
| Error | 12 | 2043.5 | 170.3 |  |  |
| Total | 14 | 5994.3 |  |  |  |

^** Statistically significant at 99% of probability level^

Table A.13: Iterated RSM design matrix for levels of pH and incubation conditions in T2

| **Run** | **pH** | | **Inoculum volume** | | **Titer (g/L)** | | **Predicted titer (g/L)** |
| --- | --- | --- | --- | --- | --- | --- | --- |
| 1 | -1 | | -1 | | 49.5 | | 40.5 |
| 2 | -1 | | 1 | | 55.3 | | 52.7 |
| 3 | 1 | | -1 | | 60.5 | | 60.9 |
| 4 | 1 | | 1 | | 62.9 | | 69.7 |
| 5 | 0 | | 0 | | 74.1 | | 74.8 |
| 6 | 0 | | 0 | | 72.3 | | 74.8 |
| 7 | 0 | | 0 | | 75.1 | | 74.8 |
| 8 | 0 | | 0 | | 74.1 | | 74.8 |
| 9 | 0 | | 0 | | 78.3 | | 74.8 |
| 10 | 0 | | -1.414 | | 59.7 | | 65.3 |
| 11 | 0 | | 1.414 | | 83.6 | | 80.1 |
| 12 | -1.414 | | 0 | | 18.3 | | 26.0 |
| 13 | 1.414 | | 0 | | 58.0 | | 52.4 |
|  |  | |  | |  | |  |
| **Levels** | | **pH** | | **Inoculum volume** | |  |  |
| 1.414 | | 6.62 | | 22.07 % | |  |  |
| 1 | | 6 | | 20 % | |  |  |
| 0 | | 4.5 | | 15 % | |  |  |
| -1 | | 3 | | 10 % | |  |  |
| -1.414 | | 2.3 | | 7.93 % | |  |  |
|  | |  | |  | |  |  |
| **Constant parameters** | | | | | |  |  |
| **Parameter** | | **Constant value** | |  | |  |  |
| Sucrose | | 20.07 %(w/v) | |  | |  |  |
| YE | | 0.0635 %(w/v) | |  | |  |  |
| (NH_4_)_2_SO_4_ | | 0.06 %(w/v) | |  | |  |  |
| K_2_HPO_4_ | | 1.5 %(w/v) | |  | |  |  |
| MgSO_4_ | | 0.02 %(w/v) | |  | |  |  |
| NaCl | | 0.1 %(w/v) | |  | |  |  |
| Incubation time | | 4 days | |  | |  |  |

Table A.14: ANOVA for quadratic model for iterated RSM for pH and incubation conditions in T2

|  | **DF** | **Sum of Squares** | **Mean Square** | **F Value** | **Prob>F** |
| --- | --- | --- | --- | --- | --- |
| pH | 1 | 699.6 | 699.6 | 17.0 | 4.00E-3** |
| Inoculum volume | 1 | 220.2 | 220.2 | 5.4 | 0.05 |
| pH.pH | 1 | 2201.0 | 2201.0 | 53.4 | 1.02E^-04^** |
| Inoculum volume.Inoculum volume | 1 | 11.6 | 11.6 | 0.3 | 0.61 |
| Inoculum volume.pH | 1 | 2.9 | 2.9 | 0.1 | 0.80 |
| Error | 7 | 288.3 | 41.2 |  |  |
| Lack of fit | 3 | 269.2 | 89.8 | 18.8 | 0.01* |
| Pure Error | 4 | 19.1 | 4.8 |  |  |
| Total | 12 | 3423.6 |  |  |  |

^** Statistically significant at 99% of probability level^

Table A.15: PBD matrix for levels of integrated factors in T3

| **Run** | **Sucrose** | **K_2_HPO_4_** | | **YE** | **(NH_4_)_2_SO_4_** | **MgSO_4_** | | **NaCl** | **pH** | **Inoculum volume** | | **Titer (g/L)** | **Predicted titer (g/L)** | |
| --- | --- | --- | --- | --- | --- | --- | --- | --- | --- | --- | --- | --- | --- | --- |
| 1 | -1 | 1 | | -1 | 1 | 1 | | 1 | -1 | -1 | | 28.0 | 32.0 | |
| 2 | -1 | -1 | | 1 | -1 | 1 | | 1 | 1 | -1 | | 5.2 | 11.3 | |
| 3 | 1 | -1 | | -1 | 1 | -1 | | 1 | 1 | 1 | | 15.5 | 21.8 | |
| 4 | -1 | 1 | | -1 | -1 | 1 | | -1 | 1 | 1 | | 17.5 | 14.7 | |
| 5 | -1 | -1 | | 1 | -1 | -1 | | 1 | -1 | 1 | | 23.0 | 32.8 | |
| 6 | 1 | -1 | | -1 | 1 | -1 | | -1 | 1 | -1 | | 18.5 | 19.3 | |
| 7 | 1 | 1 | | -1 | -1 | 1 | | -1 | -1 | 1 | | 61.5 | 57.1 | |
| 8 | -1 | 1 | | 1 | -1 | -1 | | 1 | -1 | -1 | | 26.5 | 37.0 | |
| 9 | 1 | -1 | | 1 | 1 | -1 | | -1 | 1 | -1 | | 12.5 | 17.7 | |
| 10 | -1 | 1 | | -1 | 1 | 1 | | -1 | -1 | 1 | | 34.5 | 29.6 | |
| 11 | 1 | -1 | | 1 | -1 | 1 | | 1 | -1 | -1 | | 62.5 | 53.6 | |
| 12 | -1 | 1 | | -1 | 1 | -1 | | 1 | 1 | -1 | | 8.0 | 7.0 | |
| 13 | -1 | -1 | | 1 | -1 | 1 | | -1 | 1 | 1 | | 6.4 | 8.8 | |
| 14 | -1 | -1 | | -1 | 1 | -1 | | 1 | -1 | 1 | | 29.5 | 26.0 | |
| 15 | -1 | -1 | | -1 | -1 | 1 | | -1 | 1 | -1 | | 12.5 | 10.4 | |
| 16 | 1 | -1 | | -1 | -1 | -1 | | 1 | -1 | 1 | | 78.0 | 53.5 | |
| 17 | -1 | 1 | | -1 | -1 | -1 | | -1 | 1 | -1 | | 15.5 | 12.9 | |
| 18 | -1 | -1 | | 1 | -1 | -1 | | -1 | -1 | 1 | | 20.5 | 30.3 | |
| 19 | 1 | -1 | | -1 | 1 | -1 | | -1 | -1 | -1 | | 36.0 | 42.6 | |
| 20 | 1 | 1 | | -1 | -1 | 1 | | -1 | -1 | -1 | | 53.0 | 57.1 | |
| 21 | 1 | 1 | | 1 | -1 | -1 | | 1 | -1 | -1 | | 57.0 | 56.1 | |
| 22 | -1 | 1 | | 1 | 1 | -1 | | -1 | 1 | -1 | | 7.5 | 2.9 | |
| 23 | 1 | -1 | | 1 | 1 | 1 | | -1 | -1 | 1 | | 47.0 | 42.7 | |
| 24 | 1 | 1 | | -1 | 1 | 1 | | 1 | -1 | -1 | | 33.0 | 51.1 | |
| 25 | 1 | 1 | | 1 | -1 | 1 | | 1 | 1 | -1 | | 50.5 | 34.7 | |
| 26 | 1 | 1 | | 1 | 1 | -1 | | 1 | 1 | 1 | | 34.0 | 24.5 | |
| 27 | 1 | 1 | | 1 | 1 | 1 | | -1 | 1 | 1 | | 16.5 | 23.8 | |
| 28 | -1 | 1 | | 1 | 1 | 1 | | 1 | -1 | 1 | | 21.0 | 30.4 | |
| 29 | -1 | -1 | | 1 | 1 | 1 | | 1 | 1 | -1 | | 4.5 | 2.9 | |
| 30 | -1 | -1 | | -1 | 1 | 1 | | 1 | 1 | 1 | | 7.5 | 4.5 | |
| 31 | 1 | -1 | | -1 | -1 | 1 | | 1 | 1 | 1 | | 11.5 | 32.0 | |
| 32 | 1 | 1 | | -1 | -1 | -1 | | 1 | 1 | 1 | | 21.5 | 34.5 | |
| 33 | 1 | 1 | | 1 | -1 | -1 | | -1 | 1 | 1 | | 19.9 | 30.4 | |
| 34 | -1 | 1 | | 1 | 1 | -1 | | -1 | -1 | 1 | | 27.4 | 26.1 | |
| 35 | 1 | -1 | | 1 | 1 | 1 | | -1 | -1 | -1 | | 37.7 | 42.7 | |
| 36 | -1 | -1 | | -1 | -1 | -1 | | -1 | -1 | -1 | | 27.5 | 31.9 | |
| 37 | 0 | 0 | | 0 | 0 | 0 | | 0 | 0 | 0 | | 48.5 | 29.1 | |
| 38 | 0 | 0 | | 0 | 0 | 0 | | 0 | 0 | 0 | | 46.5 | 29.1 | |
| 39 | 0 | 0 | | 0 | 0 | 0 | | 0 | 0 | 0 | | 50.5 | 29.1 | |
|  |  |  | |  |  |  | |  |  |  | |  |  | |
| **Factors** | | | **Levels** | | | | | | | | | | |  |
|  | | | Low (-1) | | | | Center (0) | | | | High (1) | | |  |
| Sucrose | | | 5 %(w/v) | | | | 12.5 %(w/v) | | | | 20 %(w/v) | | |  |
| K_2_HPO_4_ | | | 1 %(w/v) | | | | 1.5 %(w/v) | | | | 2 %(w/v) | | |  |
| YE | | | 0.03 %(w/v) | | | | 0.06 %(w/v) | | | | 0.09 %(w/v) | | |  |
| (NH_4_)_2_SO_4_ | | | 0.04 %(w/v) | | | | 0.06 %(w/v) | | | | 0.08 %(w/v) | | |  |
| MgSO_4_ | | | 0.01 %(w/v) | | | | 0.02 %(w/v) | | | | 0.03 %(w/v) | | |  |
| NaCl | | | 0.02 %(w/v) | | | | 0.26 %(w/v) | | | | 0.5 %(w/v) | | |  |
| pH | | | 4 | | | | 6 | | | | 8 | | |  |
| Inoculum volume | | | 5 % | | | | 10 % | | | | 15 % | | |  |
|  | | |  | | | |  | | | |  | | |  |
| **Constant parameters** | | | | | | | | | | | | | |  |
| **Parameter** | | | **Constant value** | | | |  | | | |  | | |  |
| Incubation time | | | 4 days | | | |  | | | |  | | |  |

Table A.16: ANOVA for linear model for PBD for integrated factors in T3

|  | **DF** | **Sum of Squares** | **Mean Square** | **F Value** | **Prob>F** |
| --- | --- | --- | --- | --- | --- |
| Sucrose | 1 | 3281.4 | 3281.4 | 24.7 | 3.02E-6** |
| K_2_HPO_4_ | 1 | 164.7 | 164.7 | 1.2 | 0.27 |
| YE | 1 | 24.0 | 24.0 | 0.2 | 0.67 |
| (NH_4_)_2_SO_4_ | 1 | 636.7 | 636.7 | 4.8 | 0.04* |
| MgSO_4_ | 1 | 28.6 | 28.6 | 0.2 | 0.65 |
| NaCl | 1 | 56.0 | 56.0 | 0.4 | 0.52 |
| pH | 1 | 4865.1 | 4865.1 | 36.5 | 1.24E-5** |
| Inoculum volume | 1 | 0.3 | 0.3 | 0.002 | 0.96 |
| Error | 30 | 3994.3 | 133.1 |  |  |
| Total | 38 | 13051.1 |  |  |  |

^* Statistically significant at 95% of probability level ** Statistically significant at 99% of probability level^

Table A.17: RSM design matrix for levels of integrated factors in T3

| **Run** | **pH** | | **Sucrose** | | **(NH_4_)_2_SO_4_** | | **Titer (g/L)** | | **Predicted titer (g/L)** |
| --- | --- | --- | --- | --- | --- | --- | --- | --- | --- |
| 1 | -1 | | -1 | | -1 | | 58.3 | | 55.9 |
| 2 | 1 | | -1 | | -1 | | 66.0 | | 65.9 |
| 3 | -1 | | 1 | | -1 | | 74.1 | | 68.7 |
| 4 | 1 | | 1 | | -1 | | 64.0 | | 63.6 |
| 5 | -1 | | -1 | | 1 | | 55.3 | | 52.2 |
| 6 | 1 | | -1 | | 1 | | 69.2 | | 71.2 |
| 7 | -1 | | 1 | | 1 | | 66.5 | | 63.1 |
| 8 | 1 | | 1 | | 1 | | 67.9 | | 66.9 |
| 9 | 0 | | 0 | | 0 | | 82.4 | | 84.3 |
| 10 | 0 | | 0 | | 0 | | 85.5 | | 84.3 |
| 11 | 0 | | 0 | | 0 | | 81.3 | | 84.3 |
| 12 | 0 | | 0 | | 0 | | 88.7 | | 84.3 |
| 13 | 0 | | 0 | | 0 | | 83.1 | | 84.3 |
| 14 | 0 | | 0 | | 0 | | 85.5 | | 84.3 |
| 15 | -1,682 | | 0 | | 0 | | 18.3 | | 25.1 |
| 16 | 1,682 | | 0 | | 0 | | 38.8 | | 36.8 |
| 17 | 0 | | -1,682 | | 0 | | 74.1 | | 74.6 |
| 18 | 0 | | 1,682 | | 0 | | 77.4 | | 81.8 |
| 19 | 0 | | 0 | | -1,682 | | 81.6 | | 84.8 |
| 20 | 0 | | 0 | | 1,682 | | 82.9 | | 84.5 |
|  |  | |  | |  | |  | |  |
| **Levels** | | **pH** | | **Sucrose** | | **(NH_4_)_2_SO_4_** | |  |  |
| 1.682 | | 7 | | 23.41 %(w/v) | | 0.049 %(w/v) | |  |  |
| 1 | | 6 | | 20 %(w/v) | | 0.04 %(w/v) | |  |  |
| 0 | | 4.5 | | 15 %(w/v) | | 0.0255 %(w/v) | |  |  |
| -1 | | 3 | | 10 %(w/v) | | 0.011 %(w/v) | |  |  |
| -1.682 | | 1.97 | | 6.59 %(w/v) | | 0.0011 %(w/v) | |  |  |
|  | |  | |  | |  | |  |  |
| **Constant parameters** | | | | | | | |  |  |
| **Parameter** | | **Constant value** | |  | |  | |  |  |
| YE | | 0.0635 %(w/v) | |  | |  | |  |  |
| K_2_HPO_4_ | | 1.5 %(w/v) | |  | |  | |  |  |
| MgSO_4_ | | 0.02 %(w/v) | |  | |  | |  |  |
| NaCl | | 0.26 %(w/v) | |  | |  | |  |  |
| Inoculum volume | | 10 % | |  | |  | |  |  |
| Incubation time | | 4 days | |  | |  | |  |  |

Table A.18: ANOVA for quadratic model for RSM for integrated factors in T3

|  | **DF** | **Sum of Squares** | **Mean Square** | **F Value** | **Prob>F** |
| --- | --- | --- | --- | --- | --- |
| pH | 1 | 165.2 | 165.2 | 9.2 | 1.28E-2* |
| Sucrose | 1 | 62.7 | 62.7 | 3.5 | 0.09 |
| (NH_4_)_2_SO_4_ | 1 | 0.1 | 0.1 | 0.0 | 0.94 |
| pH.pH | 1 | 5106.9 | 5106.9 | 283.0 | 1.16E-8** |
| Sucrose.Sucrose | 1 | 68.6 | 68.6 | 3.8 | 0.08 |
| (NH_4_)_2_SO_4_.(NH_4_)_2_SO_4_ | 1 | 0.3 | 0.3 | 0.0 | 0.90 |
| pH.Sucrose | 1 | 114.3 | 114.3 | 6.3 | 0.03* |
| pH.(NH_4_)_2_SO_4_ | 1 | 39.4 | 39.4 | 2.2 | 0.17 |
| Sucrose.(NH_4_)_2_SO_4_ | 1 | 1.9 | 1.9 | 0.1 | 0.76 |
| Error | 10 | 180.5 | 18.1 |  |  |
| Lack of fit | 5 | 144.3 | 28.9 | 4.0 | 0.08 |
| Pure Error | 5 | 36.1 | 7.2 |  |  |
| Total | 19 | 5739.9 |  |  |  |

^* Statistically significant at 95% of probability level ** Statistically significant at 99% of probability level^

Appendix B


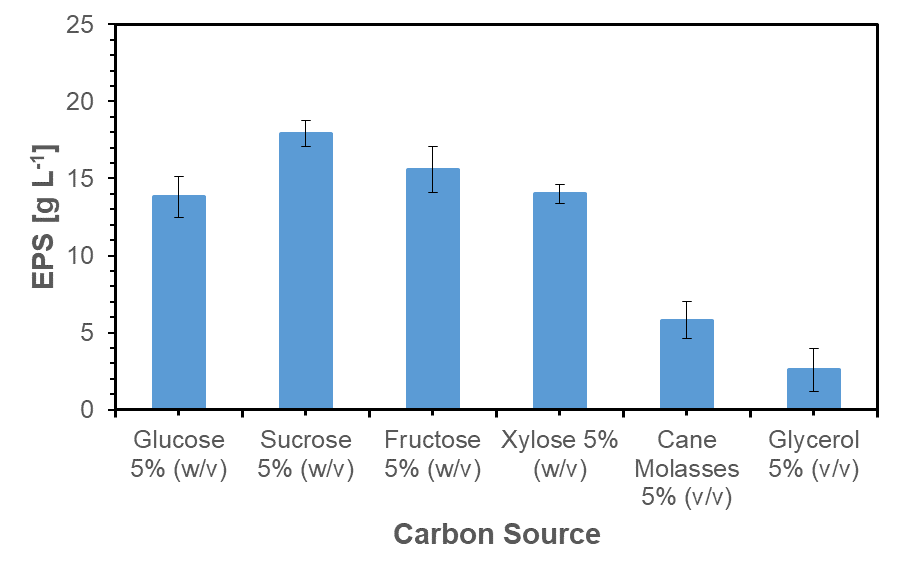


Figure B.1: Bar graph showing EPS production with different carbon sources in OFAT


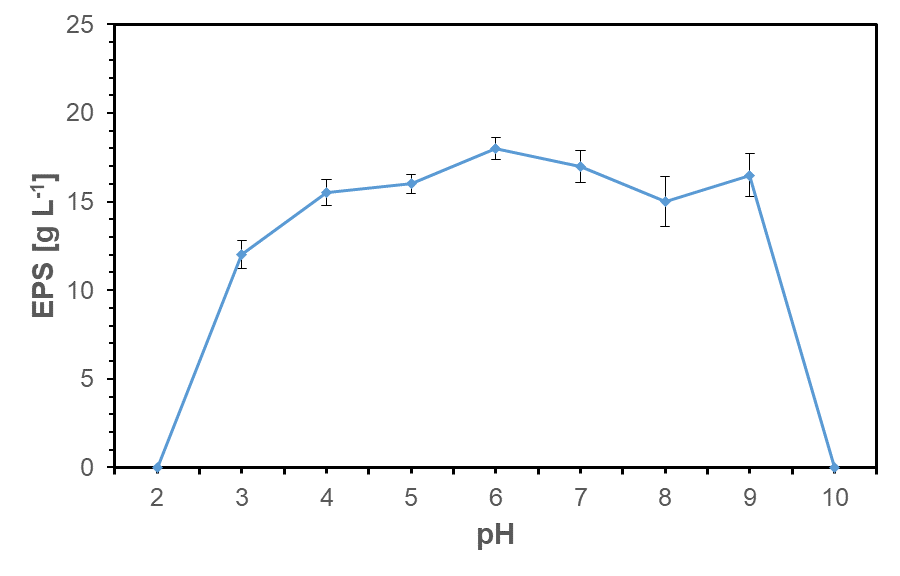


Figure B.2: Graph showing EPS production at different initial pH in OFAT


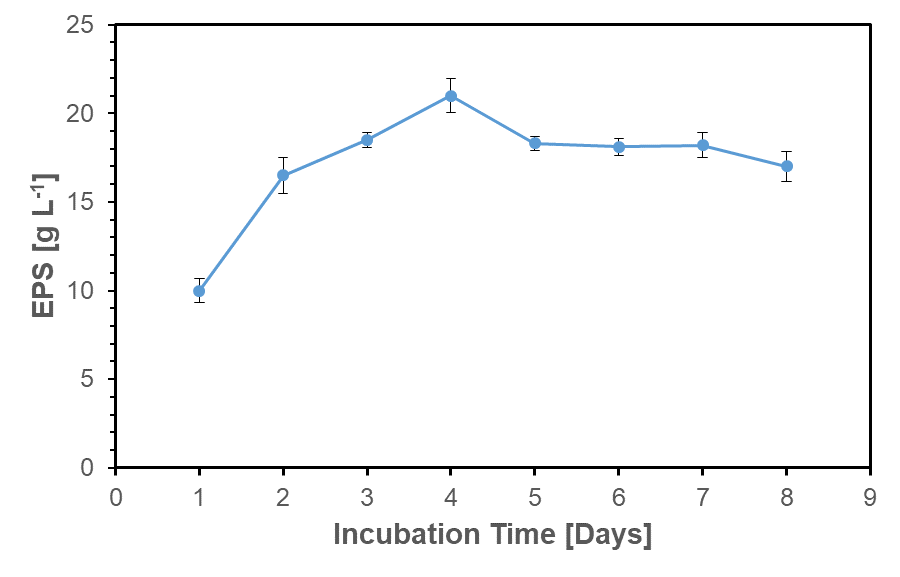


Figure B.3: Graph showing EPS production over 8 days of incubation in OFAT


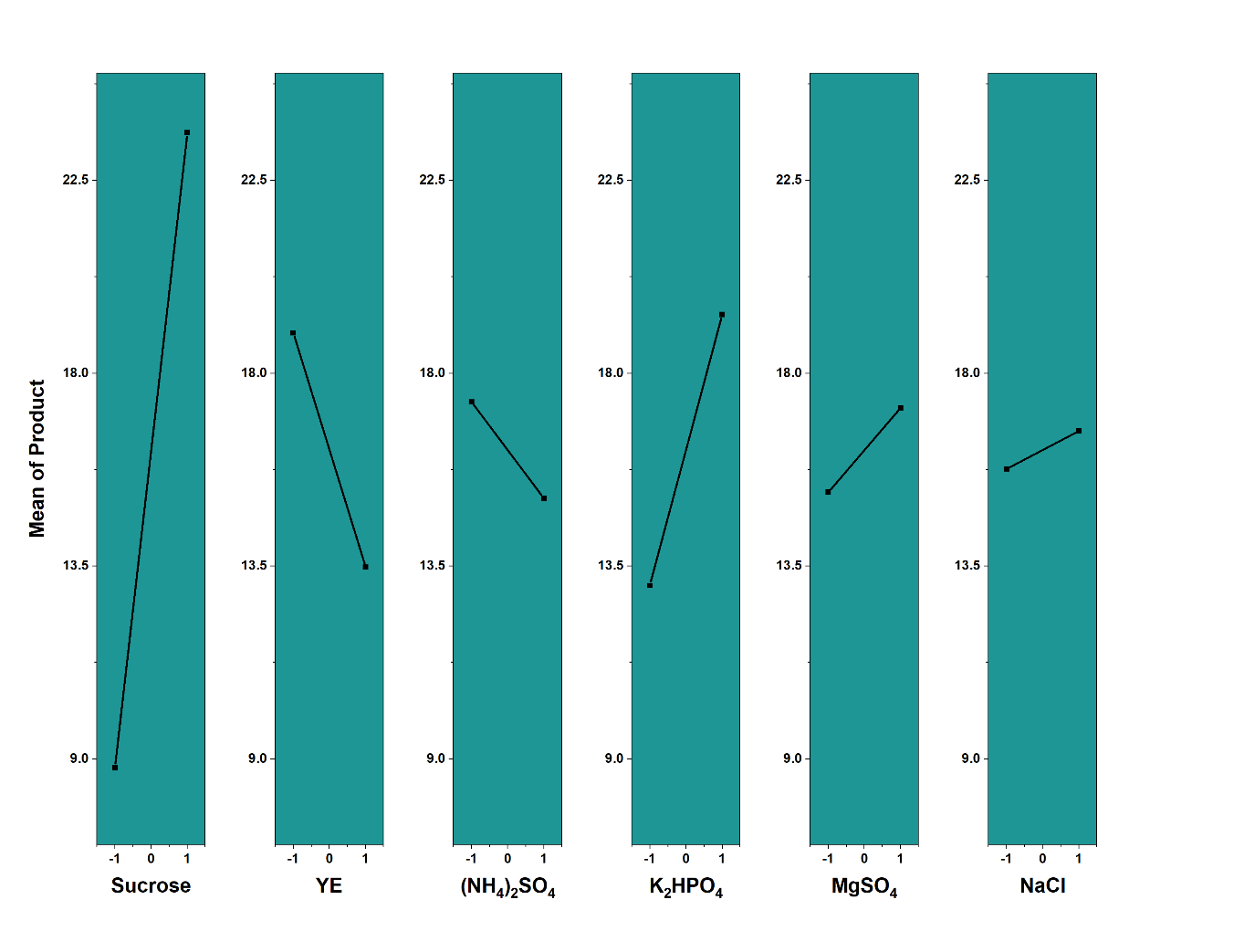


Figure B.4: Main effects plot for product for screening of significant media components in T1


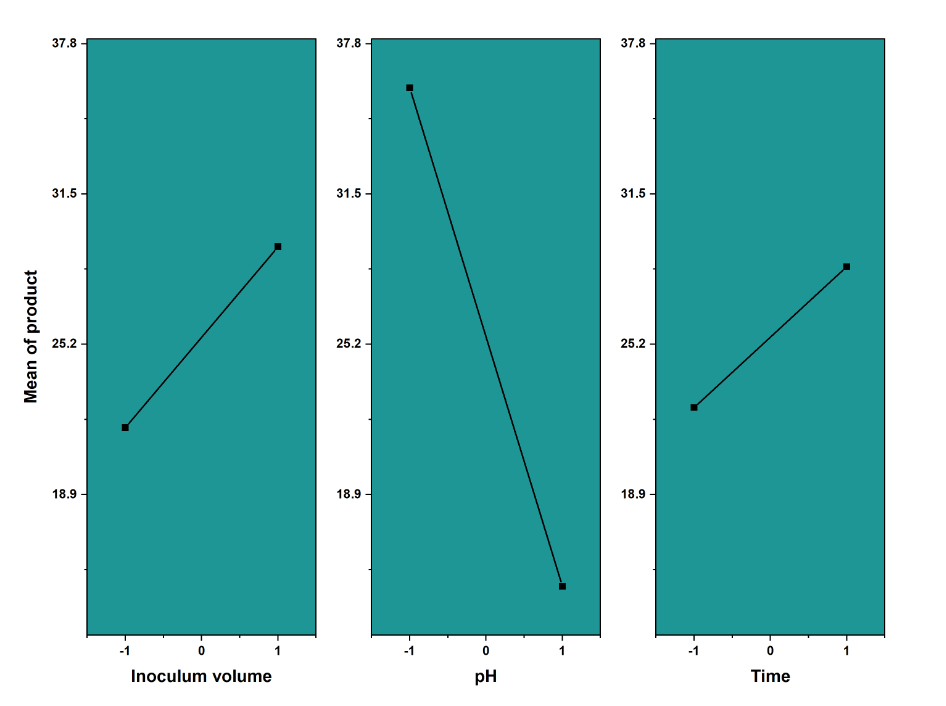


Figure B.5: Main effects plot for product for screening of significant fermentation conditions in T1


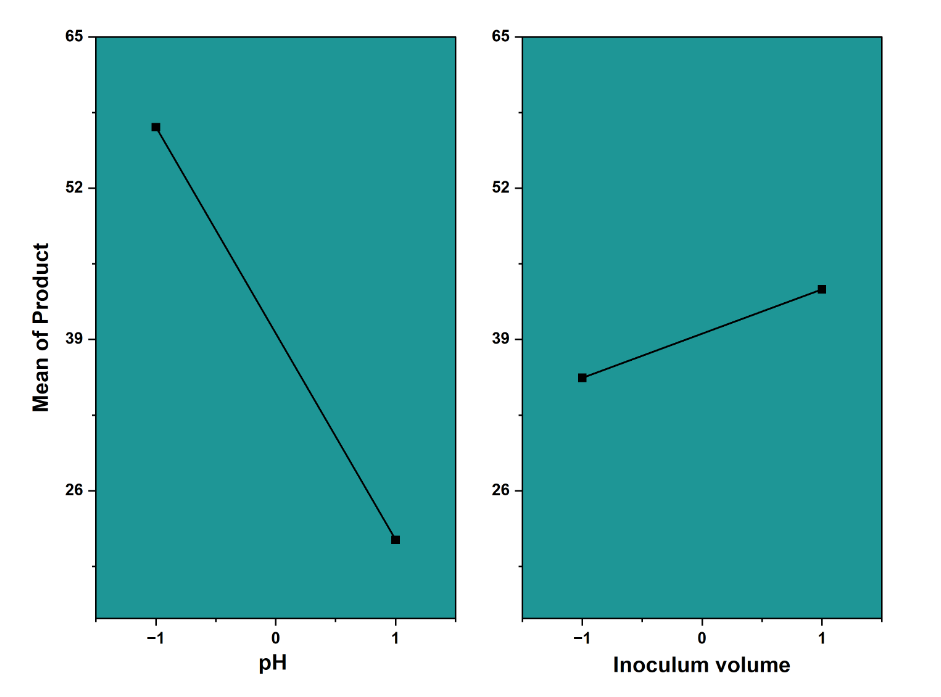


Figure B.6: Main effects plot for product for screening of significant fermentation conditions in T2


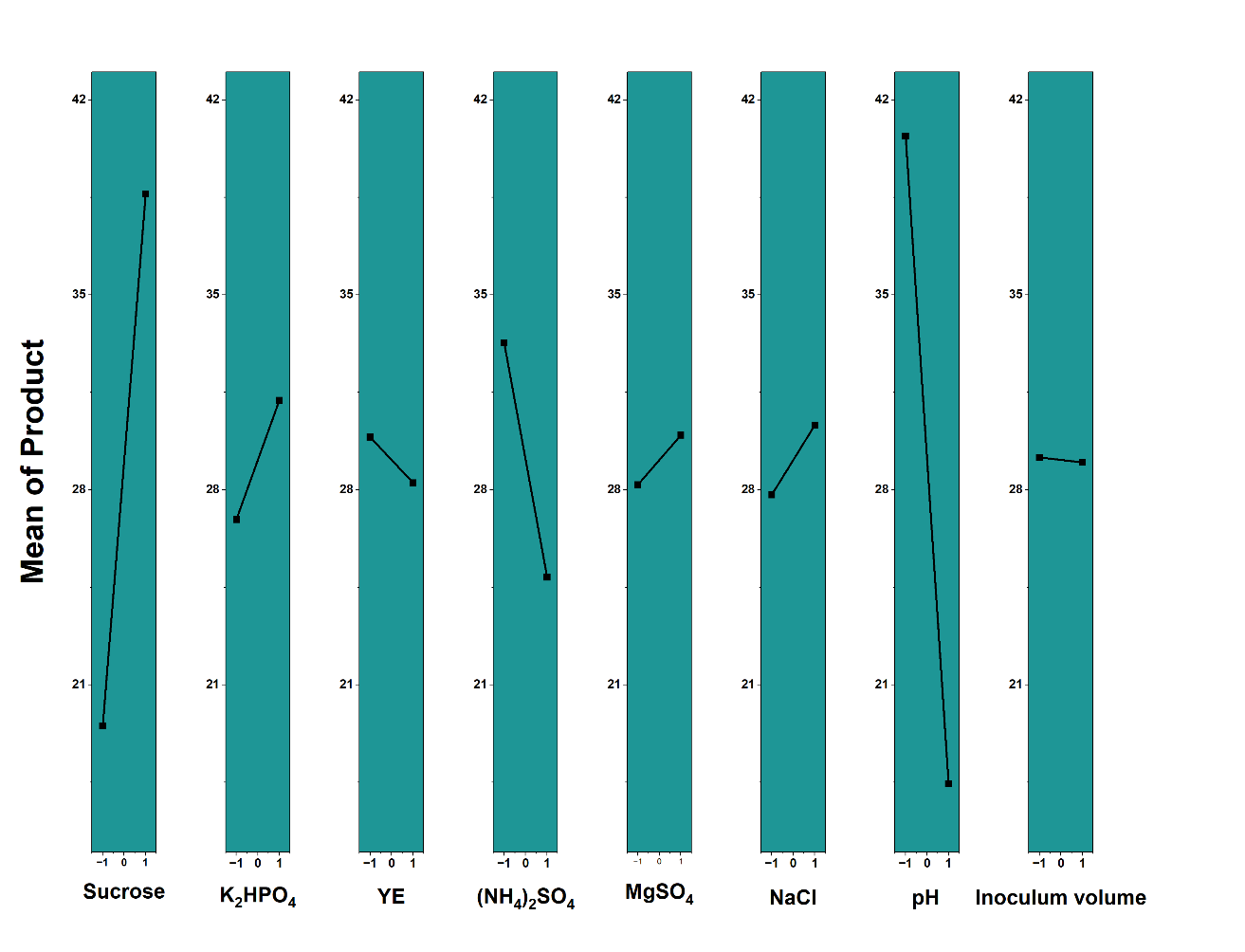


Figure B.7: Main effects plot for product for screening of significant factors in integrated PBD in T3
